# CLIC4 regulates late endosomal trafficking and matrix degradation activity of MMP14 at focal adhesions in RPE cells

**DOI:** 10.1038/s41598-019-48438-0

**Published:** 2019-08-22

**Authors:** Kuo-Shun Hsu, Wataru Otsu, Yao Li, Heuy-Ching Wang, Shuibing Chen, Stephen H. Tsang, Jen-Zen Chuang, Ching-Hwa Sung

**Affiliations:** 1 Department of Ophthalmology, Weill Medical College of Cornell University, New York, NY USA; 2 Department of Ophthalmology, Columbia University, New York, NY USA; 3 Ocular Trauma Task Area, US Army Institute of Surgical Research, Joint Base San Antonio-Fort Sam Houston, TX San Antonio, USA; 4 Department of Surgery and Department of Biochemistry, Weill Medical College of Cornell University, New York, NY USA; 5 Institute of Human Nutrition, Vagelos College of Physicians and Surgeons, Columbia University, New York, NY USA; 6 Jonas Children’s Vision Care and Bernard & Shirlee Brown Glaucoma Laboratory, Edward S. Harkness Eye Institute, New York-Presbyterian Hospital, New York, NY USA; 7 Department of Pathology & Cell Biology, and Columbia Stem Cell Initiative, Columbia University Medical Center, New York, NY USA; 8 Department of Cell and Developmental Biology, Weill Medical College of Cornell University, New York, NY USA; 9 Present Address: Department of Surgery, Colorectal Service and Laboratory of Signal Transduction, Memorial Sloan Kettering Cancer Center, New York, NY USA; 10 Present Address: Department of Biomedical Research Laboratory, Gifu Pharmaceutical University, Gifu, Japan

**Keywords:** Biochemistry, Membrane trafficking

## Abstract

Dysregulation in the extracellular matrix (ECM) microenvironment surrounding the retinal pigment epithelium (RPE) has been implicated in the etiology of proliferative vitreoretinopathy and age-related macular degeneration. The regulation of ECM remodeling by RPE cells is not well understood. We show that membrane-type matrix metalloproteinase 14 (MMP14) is central to ECM degradation at the focal adhesions in human ARPE19 cells. The matrix degradative activity, but not the assembly, of the focal adhesion is regulated by chloride intracellular channel 4 (CLIC4). CLIC4 is co-localized with MMP14 in the late endosome. CLIC4 regulates the proper sorting of MMP14 into the lumen of the late endosome and its proteolytic activation in lipid rafts. CLIC4 has the newly-identified “late domain” motif that binds to MMP14 and to Tsg101, a component of the endosomal sorting complex required for transport (ESCRT) complex. Unlike the late domain mutant CLIC4, wild-type CLIC4 can rescue the late endosomal sorting defect of MMP14. Finally, CLIC4 knockdown inhibits the apical secretion of MMP2 in polarized human RPE monolayers. These results, taken together, demonstrate that CLIC4 is a novel matrix microenvironment modulator and a novel regulator for late endosomal cargo sorting. Moreover, the late endosomal sorting of MMP14 actively regulates its surface activation in RPE cells.

## Introduction

The extracellular matrix (ECM) is a highly dynamic structure that continuously undergoes regulated remodeling^1^. Matrix metalloproteinase 14 (MMP14 or MT1-MMP) is one of the best studied membrane-type matrix metalloproteinases. MMP14 proteolyzes a variety of ECM components (e.g., gelatin, fibronectin, collagen) at pericellular sites^2,3^ and plays critical roles in tissue homeostasis during normal development (e.g., angiogenesis, renal tubulogenesis) and pathological conditions (e.g., tumor invasion)^4–8^. The precise execution of MMP14-mediated matrix digestion relies on its tightly regulated surface expression and several context-dependent factors (e.g., autocatalytic inactivation, ectodomain shedding, dimerization, inhibitor binding)^9–13^. Studies in cancer cell lines have long shown that the surface expressed MMP14 is predominantly enriched in the invadopodia (or podosomes). Invadopodia are short-lived, F-actin-rich circular-shaped cell protrusions that typically formed at the central region of the cells^14,15^. More recently, active MMP14 is also found to be expressed in focal adhesions^16–18^, a structure which has been better known as a cell-substratum contact involving cell migration. MMP14 is also known to regulate the activation of other MMPs. Most notably, surface MMP14 proteolytically cleaves Pro-MMP2 and releases active MMP2 into the extracellular milieu for ECM remodeling^19^.

Cancer cell studies showed that the surface expression of MMP14 is highly dynamic and regulated through the endocytic trafficking pathway involving the late endosome (LE)^20–22^. The LE has now emerged as an important sorting station, beyond its original role as a lysosomal precursor^23,24^. The LE vacuole has an acidic pH and contains multiple intraluminal vesicles. The majority of MMP14 internalized from the cell surface resides in the LE^25^. The LE-expressed MMP14 molecules are recycled back to the cell surface via the recycling endosome, through a process involving chloride intracellular channel 3 (CLIC3)^21,22,26^.

CLIC3 belongs to an evolutionarily conserved CLIC family proteins (CLIC1-CLIC6). They are so named because several CLICs generate chloride conductance in biological and artificial membranes^27^. CLIC4 is one of the better characterized CLICs. Our lab previously showed that CLIC4 deficient mice had dysregulated renal tubulogenesis^28^, a phenotype shared by the MMP14 deficient mice^29^. Furthermore, the expression of CLIC4 is susceptible to environmental changes (e.g., oxidative stress, transforming growth factors, lipopolysaccharide, nitric oxide) that are also known to modulate the expression level of MMP14^30–37^.

These coincidences prompted us to investigate the putative relationship between CLIC4 and MMP14 in retinal pigment epithelium (RPE) cells. In the retina, RPE cells form a polarized monolayer surrounded by the ECM, which serves as an interface between the neural retina and the choroid. The knockdown (KD) of CLIC4 in RPE cells *in vivo* disrupts the membrane specializations (apical microvilli, basal infoldings) of these cells^38^. Since the morphogenesis and maintenance of these membrane structures is coupled to the homoeostasis of surrounding ECMs, the above *in vivo* observation suggests the involvement of the RPE-expressed CLIC4 in ECM remodeling. The concomitant dysregulation in the membrane specialization and ECM homeostasis of the RPE has been widely implicated in the pathogenesis of proliferative vitreoretinopathy^39^ and age-related macular degeneration (AMD)^40–42^. AMD is the leading cause of vision loss in elderly people. While deciphering how ECM remodeling affects the progression of these diseases may lead to new therapies, the molecular dissection and regulation of the matrix remodeling function of RPE cells is challenging due to their complex cell-cell and cell-matrix interactions. The gelatinase activity of the MMP2 secreted from the MMP14-overexpressing human ARPE19 cells and from human RPE monolayers has been studied using zymography assays^43–47^. The pericellular ECM degradation function of the endogenous MMP14 in RPE cells and its regulatory pathway, however, have not been investigated. In the present paper, we employed the cell-based matrix degradation assay in ARPE19 cells. We show that the focal adhesions are the degradation foci of these cells. MMP14 and CLIC4 both have an important role in the dynamic ECM remodeling of the ARPE19 cells. Mechanistically, CLIC4 regulates the matrix degradation activity of MMP14 by controlling its proper LE sorting and proteolytic activation in lipid rafts. Corroborating with CLIC4’s role in regulating the ECM remodeling, we demonstrated that in polarized human RPE monolayers, the secretion of MMP2 was significantly reduced when CLIC4 was suppressed.

## Results

### Focal adhesions act as the ECM degradation foci of RPE cells

**Figure 1:**
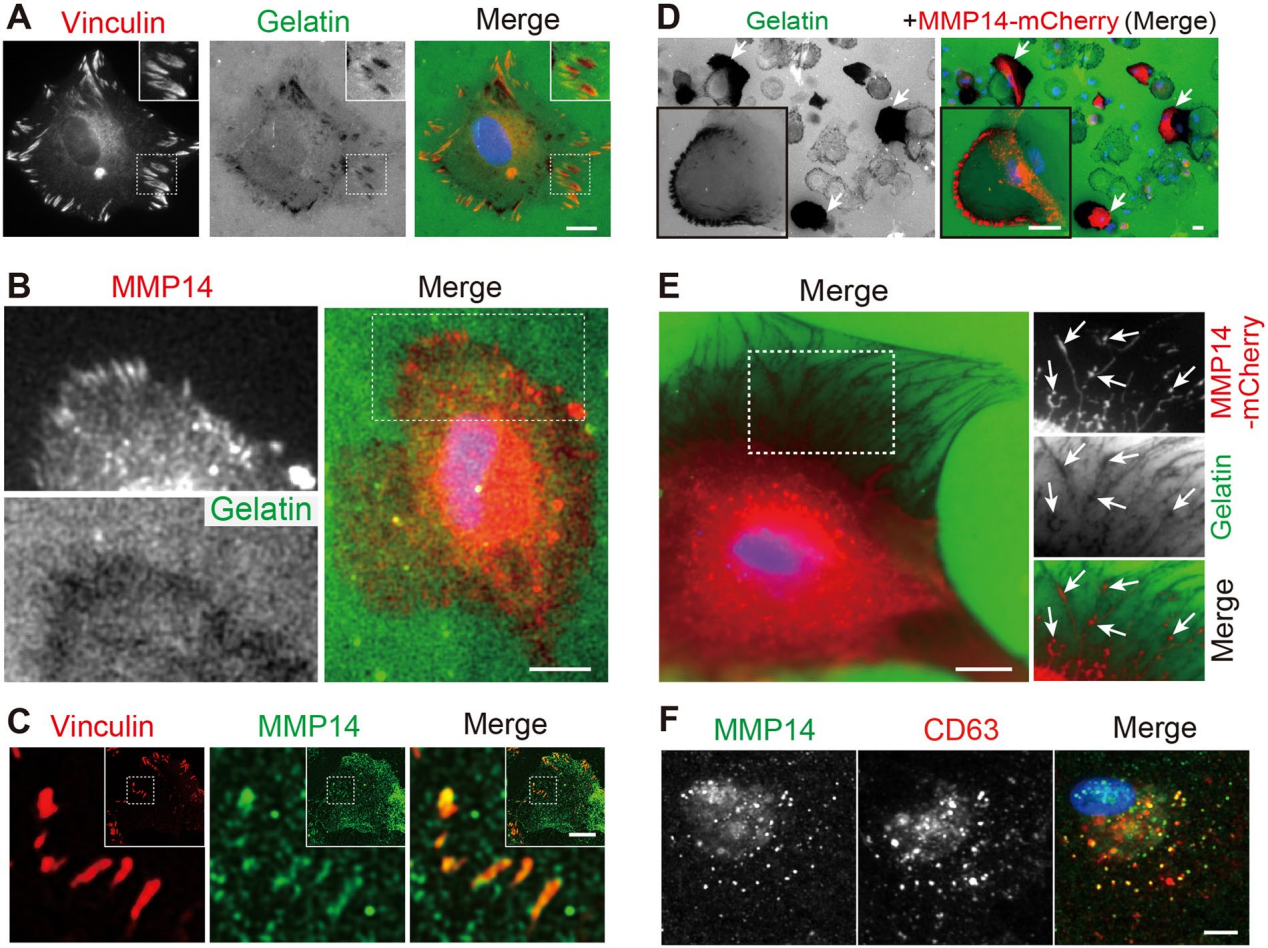
MMP14 expression in degradative focal adhesions in RPE cells. (**A**,**B**) Representative images of ARPE19 plated on a fluorescein-conjugated gelatin coverslip for 5 hours and immunostained with anti-vinculin (in **A**) or anti-MMP14 (in **B**) antibodies followed by Alexa 568-secondary antibodies. Black-and-white single-channel images and the merged color images are shown. Enlarged views are from the boxed areas showing the overlapping vinculin signal and degradation foci. (**C**) Representative low-power (insets) and high-power images of ARPE19 cells plated on non-fluorescent gelatin-coated coverslips for 5 hours and labeled for MMP14 (green) and vinculin (red). (**D**,**E**) ARPE19 cells transfected with MMP14-mCherry for one day were plated on fluorescein-conjugated gelatin coverslips for 5 hours. Both low-power (**D**) and high-power (**E**) views are shown. Arrows in (**D**) point to the cells with massive gelatin degradation activity caused by the ectopic expression of MMP14-mCherry. Arrows in (**E**) point to the MMP14-mCherry-labeled tubulovesicles that match the gelatin degradation footprints. (**F**) Representative images of ARPE19 cells plated on non-coated coverslips and immunostained for endogenous MMP14 (green) and CD63 (red). Blue (in **A**,**B**,**D**–**F**): DAPI nuclear stain. Scale bars (in **A**–**F**) = 10 µm.

To investigate ECM degradation, we subjected ARPE19 cells to a gelatin degradation assay commonly used for cancer cells. In this assay, the cell surface localized MMP cleaves the fluorescein-gelatin matrix coating underneath the cell, leaving “dark” footprints behind before the cells migrate away. These experiments showed that, at 5 hours after plating, ARPE19 cells produced oblong-shape, degradation foci predominantly located at the cell periphery (Fig. 1A). The morphology and the distribution of the degradation foci resembled those of focal adhesions. Indeed, the staining of the focal adhesion marker vinculin shared a similar pattern and a partial overlap with the gelatin-degradation foci (Fig. 1A).

Unlike ARPE19 cells, MDA-MB-231 cancer cells generated circular-shape dark footprints on the gelatin coated coverslips (Fig. S1A), likely through the invadopodia described in the literature^14,15^. The degradation foci of MDA-MB-231 cells were not coincided with the vinculin-rich focal adhesion structures (Fig. S1A). Invadopodia are actin-rich circular-shape structures^14,15^. While weak F-actin puncta were found in the ARPE19 cells, these structures did not share any specific associations with the gelatin degradation foci (Fig. S1B).

### MMP14 mediates the matrix degradation of RPE cells at focal adhesions

Several pieces of evidence collectively suggested that MMP14 importantly contributes to the ECM digestion at focal adhesions in RPE cells. Firstly, endogenous MMP14 was frequently associated with both the gelatin degradation foci (Fig. 1B) and vinculin-labeled focal adhesions (Fig. 1C). Secondly, the ARPE19 cells transfected with MMP14-mCherry (Fig. 1D), but not mCherry alone (Fig. S1C), had markedly enhanced gelatin degradation activity compared to the neighboring non-transfected cells. Although it was difficult to clearly identify the degradation foci in the high expressers, in the slightly lower expressers, the expression pattern of the MMP14-mCherry was in a good correlation with that of the gelatin degradation, implying that the MMP14 expressed in ARPE19 cells is proteolytically active. Also, as previously described^25^, the transfected MMP14-mCherry was detected on both the LE-like granular structures and the transporting tubules. The colabeling of the endogenous MMP14 and CD63 (a LE marker) confirmed that at the steady state a large subset of the MMP14 was expressed in the LE (Fig. 1F; Pearson’s correlation coefficient = 0.52 ± 0.08; n = 28 cells).

**Figure 2:**
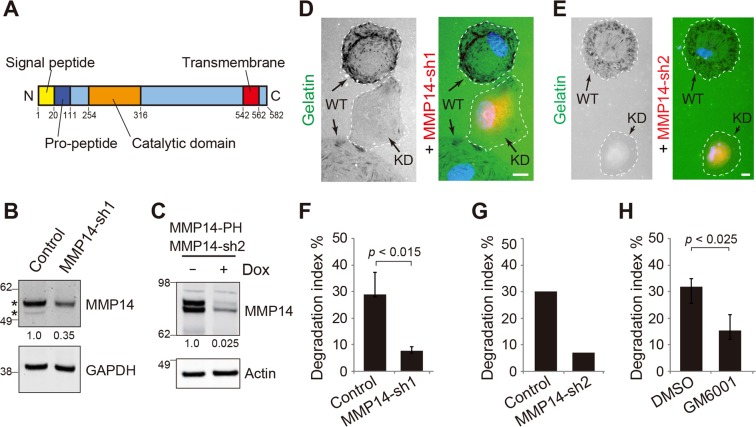
MMP14 mediates matrix digestion in RPE cells. (**A**) A schematic diagram depicting the motifs contained within MMP14. (**B**) Immunoblots of lysates of ARPE19 cells transiently transfected with or without MMP14-sh1 plasmid and probed with anti-MMP14 or anti-GAPDH antibodies. Both the ~57-kDa and the ~53-kDa bands (asterisks) detected by anti-MMP14’s N-terminus antibody were reduced in the MMP14-sh1 transfected cells, indicating that they were differently processed forms of MMP14. The relative MMP14 signal intensity is also shown. (**C**) Immunoblots of 293 T cell lysates containing 1 day-transfected MMP14-PHluorin (PH) reporter and MMP14-sh2/turboRFP, treated with (+) or without (−) Dox, and probed by the indicated antibodies. The estimated KD effect is also shown. (**D**,**E**) ARPE19 was transfected with MMP14-sh1/mCherry (**D**) or MMP14-sh2/turboRFP (**E**) for 3 days and plated on a fluorescein-conjugated gelatin coverslip for 5 hours. The representative images show that the “red” CLIC4-KD cells had less gelatin degraded footprints compared to the adjacent two non-transfected (WT) control cells. The cell borders were marked by the dashed lines. Blue: DAPI nuclear staining. Scale bar = 10 µm. (**F**) Quantification of gelatin degradation assay of non-transfected control cells or “red” cells expressing either MMP14-sh1/mCherry (**F**) or MMP14-sh2/turboRFP (**G**). The y-axis of the histograms shows the percentage of the cells that are active in gelatin degradation (i.e., possessing >10 clear dark foci within the cell or at the cell periphery). For (**F**) n > 160 cells for either transfected cells or non-transfected neighboring (control) cells; three independent replicates. Error bars, standard deviation. p value, t-test. N = 3. For (**G**) n = 70 cells for control cells; n = 28 cells for MMP14-sh2/turboRFP cells. N = 1. (**H**) Quantification of gelatin degradation assays of naïve ARPE19 cells treated with 0.1% DMSO or 100 nM GM6001. n > 500 cells for each sample per test. The graphs represent means ± S.D. of three independent tests. p value, student’s t-test.

Thirdly, the ARPE19 cells with MMP14 suppressed had significantly fewer gelatin degradation foci. In these experiments, two short-hairpin RNAs (sh1, sh2) that targeted two distinct sequences of MMP14 were employed. The MMP14-sh1 was directed by the U6 promoter and was constitutively expressed upon transfection. We performed immunoblotting assays to confirm the knockdown (KD) effect by utilizing an antibody that recognized the N-terminus (amino acids 150–180; Fig. 2A) of MMP14. We detected a major ~57-kDa band (representing the mature membrane tethered form), and a minor ~53-kDa band (presumably with cleaved cytoplasmic tail^9^) in control cells (Fig. 2B). The MMP14-sh1 transfected ARPE19 cells had both of these MMP14 species detectably reduced (Fig. 2B). The MMP14-sh2 was directed by the rtTA3/tetracycline operator-miniCMV controlled promoter and was only expressed upon induction by doxycycline (Dox). We verified the silencing of MMP14-sh2 using transfected MMP14-PHluorin reporter. Immunoblotting assays showed that the expression of MMP14-PHluorin was quantitatively reduced in Dox-treated MMP14-sh2 transfected cells (Fig. 2C). In order to identify the MMP14-KD cells in the gelatin degradation assays without ambiguity, we inserted a red fluorescent reporter protein in the plasmid that encoded MMP14-sh (mCherry for MMP14-sh1, and turboRFP for MMP14-sh2). The “red” ARPE19 cells, transfected with either MMP14-sh1 (Fig. 2D,F) or MMP14-sh2 (Fig. 2E,G), showed significantly fewer cells had “dark” gelatin degradation foci.

Fourthly, we showed that GM6001 (a broad spectrum metalloprotease inhibitor that also inhibits MMP14’s activity^48^), but not DMSO control, significantly reduced the gelatin degradative activity of ARPE19 cells (Fig. 2H). The above results collectively suggest that MMP14 importantly contributes to the ECM digestion at focal adhesions in ARPE19 cells. Furthermore, the partial overlap between the degradation foci, MMP14, and vinculin indicates that the focal adhesion expression of the active MMP14 is dynamically regulated (Fig. 1A–C).

### CLIC4 regulates the matrix degradation of focal adhesions in RPE cells

**Figure 3:**
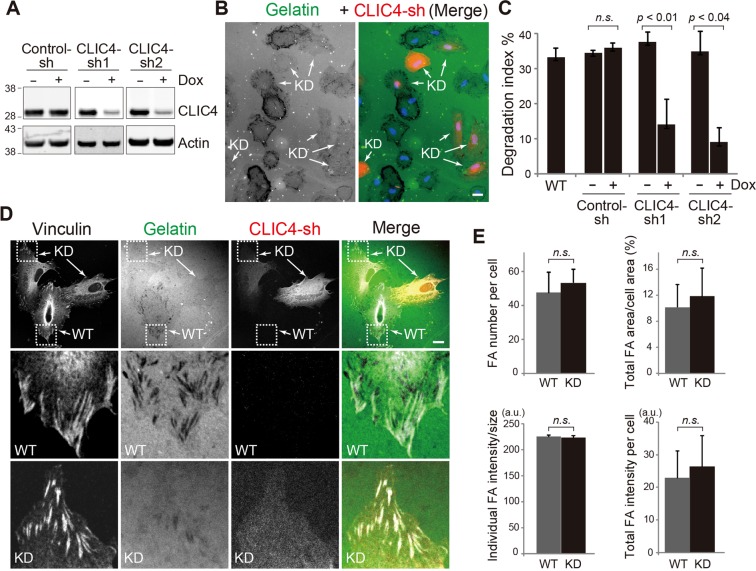
CLIC4 regulates matrix digestion at focal adhesions in ARPE19 cells. (**A**) Immunoblotting of ARPE19 lines stably expressing Dox-regulated control-sh, CLIC4-sh1, or CLIC4-sh2. Cells were treated with (+) or without (−) Dox for 3 days before the cell lysates were harvested for immunoblotting using anti-CLIC4 and anti-actin antibodies. (**B**) ARPE19 cells stably expressing inducible CLIC4-sh1/turboRFP, either treated with or without Dox, were mixed and plated on the same fluorescein-conjugated gelatin coverslips for 5 hours. Representative single-channel view of gelatin (in black and white) and merged color image are shown. The “red” CLIC4-KD cells clearly had reduced gelatin degradation activity. (**C**) Quantification of gelatin degradation activity of Dox treated (+) or untreated (−) ARPE19 cells stably transfecting control-sh, CLIC4-sh1, or CLIC4-sh2 as well as the naïve (“WT”) ARPE-19 cells. N > 400 for each sample, 3 repeats. Error bars, standard deviation. p, t-test; n.s., not significant. (**D**) A mixture of ARPE19 with (i.e., KD) or without (i.e., WT) CLIC4 suppressed as described in (**B**) were plated together on the fluorescein-conjugated gelatin coverslip for 5 hours, and immunostained with vinculin. The bottom two panels show enlarged views of the boxed areas in the top panels. (**E**) Histograms show four different parameters of evaluating the effect of FA assembly by CLIC4 KD using vinculin immunolabeling described in D. n = 435 foci from 8 “red” CLIC4-KD cells. n = 343 foci from 6 control “WT” cells. The graphs in (**C**,**E**) represent means ± S.D. of three independent tests. p value, student’s t-test. Scale bars (in **B**,**D**) = 10 µm.

We set out to examine the role CLIC4 plays in the gelatin degradation of RPE cells. To avoid long-term CLIC4 silencing, we generated ARPE19 lines stably expressing Dox-induced CLIC4-sh/turboRFP. The level of endogenous CLIC4 was reduced to ~20–30% in the lines expressing two different CLIC4-sh (sh1, sh2), but not the scrambled control-sh, upon 3-day Dox treatment (Fig. 3A). For gelatin degradation assays, we plated equal numbers of Dox-treated and Dox-untreated cells on the same fluorescein-gelatin coated coverslips. The “red” CLIC4-KD cells had significantly reduced matrix proteolysis (Figs. 3B,C). In contrast, regardless of Dox treatment, the control-sh expressing RPE cells had similar gelatin digestion as the naïve “wild type” (WT) RPE cells (Fig. 3C).

The expression level of CLIC4 was shown to affect the focal adhesion formation in cancer cell lines (HeLa and MDA-MB-231)^49^. Thus, we examined whether the reduced gelatin degradation seen in the CLIC4-KD ARPE19 cells was due to decreased focal adhesion formation. Strikingly, while the gelatin digestion was largely compromised in “red” CLIC4-KD cells, the focal adhesions of these cells formed normally (Fig. 3D). The quantifications demonstrated that the number of focal adhesions, the vinculin labeling intensity, and the average size of focal adhesions of the CLIC4-KD cell were indistinguishable from those of control cells (Fig. 3E). Collectively, these data suggest that CLIC4 is required for MMP14-mediated pericellular ECM proteolysis at focal adhesions through a mechanism independent of focal adhesion assembly.

### CLIC4 regulates the lipid raft expression of MMP14

**Figure 4:**
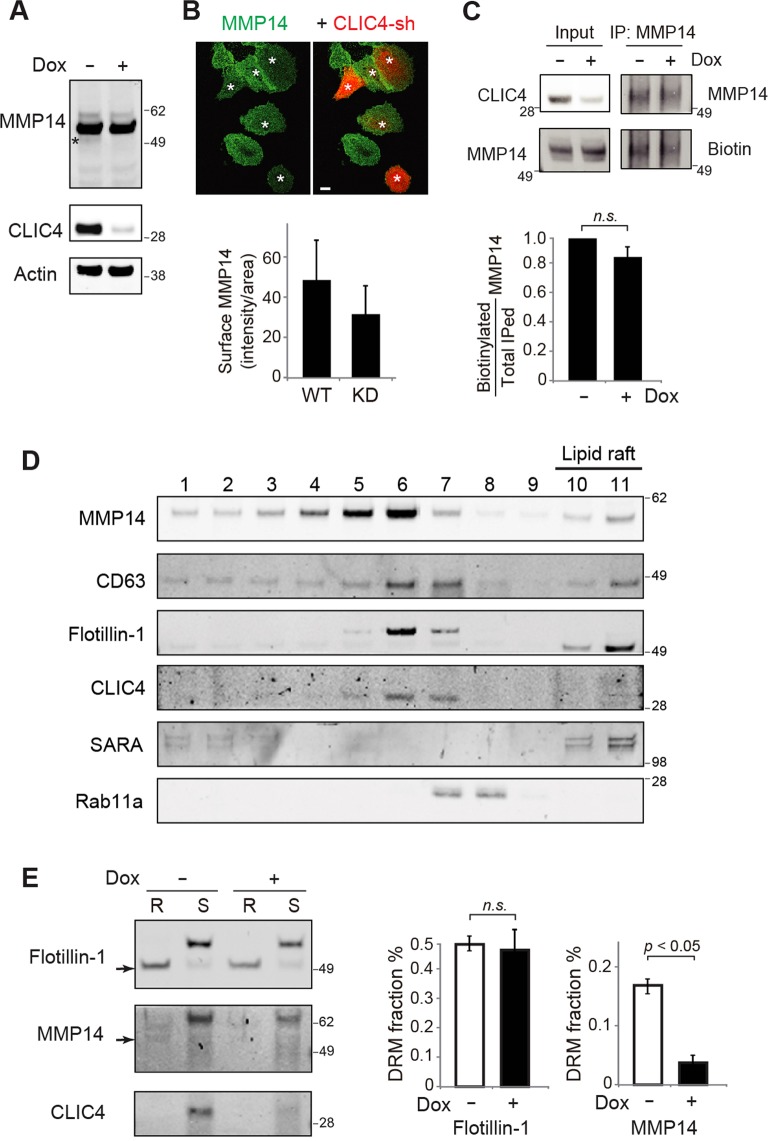
CLIC4 regulates lipid raft expression, but not cell surface expression, of MMP14 in ARPE19 cells. (**A**) Representative immunoblots of ARPE19 lysates stably expressing inducible CLIC4-sh/turboRFP, treated with (+) or without (−) Dox for three days. Dox treatment drastically reduced the expression level of CLIC4 but not MMP14 except the 53-kDa species (asterisk), which became less detectable. (**B**) A representative image of a mixture of ARPE19 cells stably expressing inducible CLIC4-sh/turboRFP, which were treated with Dox not, were stained with an antibody recognizing the extracellular domain of MMP14 under non-cell permeabilized conditions from three independent experiments. Scale bar = 10 µm. Asterisks marked the transfected cells that displayed the red fluorescence. The histogram shows the MMP14 signal density (intensity/surface area) of 37 cells for “WT” and 25 cells for CLIC4-KD. (**C**) Surface biotinylation assays of inducible CLIC4-sh1 expressing ARPE19 cells, treated with or without Dox. Total cell lysates (input) and MMP14 immunoprecipitates were immunoblotted with the indicated antibodies or streptavidin for biotin detection. A histogram shows the ratios of surface expressed MMP14 relative to the total immunoprecipitated MMP14. (**D**) Immunoblots of 5%-20% sucrose fractionations of TritonX-100 lysates of naïve ARPE19 cells probed with indicated antibodies. The light, detergent-insoluble fractions (#10, #11) are considered to contain lipid rafts. (**E**) (Left) ARPE19 cells stably expressing inducible CLIC4-sh/turboRFP, Dox treated or not, were subjected to detergent-resistant membrane partition analysis. The detergent-soluble (S) and detergent-resistant (R) fractions were immunoblotted. Arrows point to the smaller molecular mass, cleaved species of Flotillin-1 and MMP14. (Right) A histogram shows the relative fraction of cleaved forms of Flotillin-1 (49-kDa) and MMP14 (~53-kDa) into the detergent-resistant membranes (DRM). Y-axis shows the ratio of [the signal intensity of lower molecular mass species in the R fraction]/[the signal intensity of both low and high molecular mass species in both R and S fraction]. KD of CLIC4 by Dox (+) significantly reduced the expression of the proteolytic form of MMP14, but not Flotillin-1, in the detergent-resistant membrane fractions. Bars show means ± S.D. from three independent experiments. p value, t-test. n.s.: not significant.

We set out to examine the mechanism by which CLIC4 regulates the gelatin degradation of ARPE19 cells. Using immunoblotting assays, we first showed that, at steady state, the total expression level of the main species (~57 KDa) of MMP14 was unaffected by the reduced CLIC4. The ~53-kDa species was hard to see in the CLIC4-KD cells (Fig. 4A). We then performed surface immunostaining of MMP14 in non-permeabilized cells using an antibody that recognizes the extracellular domain of MMP14 (Fig. 4B). We employed a mixture of equal numbers of ARPE19 cells, with or without CLIC4 suppressed. The quantification of the density of the surface MMP14 signal showed no statistically significant difference between “red” CLIC4-KD cells and the neighboring non-transfected cells, albeit there was heterogeneity among the cells. To corroborate this observation biochemically, we used surface biotinylation assays to confirm that the amount of total surface-expressed MMP14 was similar between the control and the CLIC4-KD cells (Fig. 4C).

MMP14 is known to be expressed in detergent-resistant cholesterol-rich membrane microdomains (i.e., lipid rafts). The lipid raft-expressed MMP14, dominated by the C-terminal processed form(s), serves as the major form that is responsible for ECM degradation in cancer cells^9,50–53^. The above observations prompted us to ask whether CLIC4 affects the lipid raft expression of MMP14 in ARPE19 cells. To this end, we performed sucrose gradient fractionation of Triton X-100 lysates of naïve ARPE19 cells. These fractions were probed with flotillin-1, which is commonly regarded as a lipid-raft marker that is dynamically expressed in both LEs and plasma membranes^54,55^. These fractions were also probed with CLIC4, CD63, the early endosome marker SARA^56^, and the recycling endosome marker Rab11a (Fig. 4D). In the soluble fractions (#1–#9), the MMP14 signal peaked in fraction #6, which also contains the peak signals of CD63, flotillin1 and CLIC4. MMP14 had little or no overlap with SARA in the soluble fractions and a partial overlap with Rab11 in fraction #7.

MMP14, CD63 and flotillin-1 were also detected in the TritonX-100 insoluble fraction that floated to the low-density fractions (#10, #11), i.e., the lipid rafts. As previously reported^51^, MMP14 in the lipid fractions had a smaller molecular mass compared to its counterpart in the heavier fractions (~53-kDa vs. ~57 kDa) (Fig. 4D). The flotillin-1 in the light fractions had the predicted molecular mass of ~49-kDa, and curiously, it also ran slightly faster than its counterparts in the heavier fractions.

To measure the lipid raft partitioning change of MMP14 affected by CLIC4, we compared the MMP14 expression in the detergent-soluble vs. detergent-resistant membrane fractions of ARPE19 cells with or without suppression of CLIC4 by Dox. The quantification of three independent assays showed that the relative partition of the low molecular mass species of MMP14 to the detergent-resistant membrane fraction was significantly reduced in CLIC4-KD cells (Fig. 4E). In contrast, the relative partition of flotillin-1 to the detergent-resistant pool was indistinguishable between control and CLIC4-KD cells (Fig. 4E). The partition of the high molecular mass species of MMP14 and that of the MMP14 to the soluble pool was, however, not significantly altered by CLIC4 silencing (not shown). These results support the idea that while CLIC4 is not critical for the lipid raft genesis per se, it selectively modulates the lipid raft expression of the proteolytically active form of MMP14.

### LE expression of CLIC4 and its interaction with MMP14

**Figure 5:**
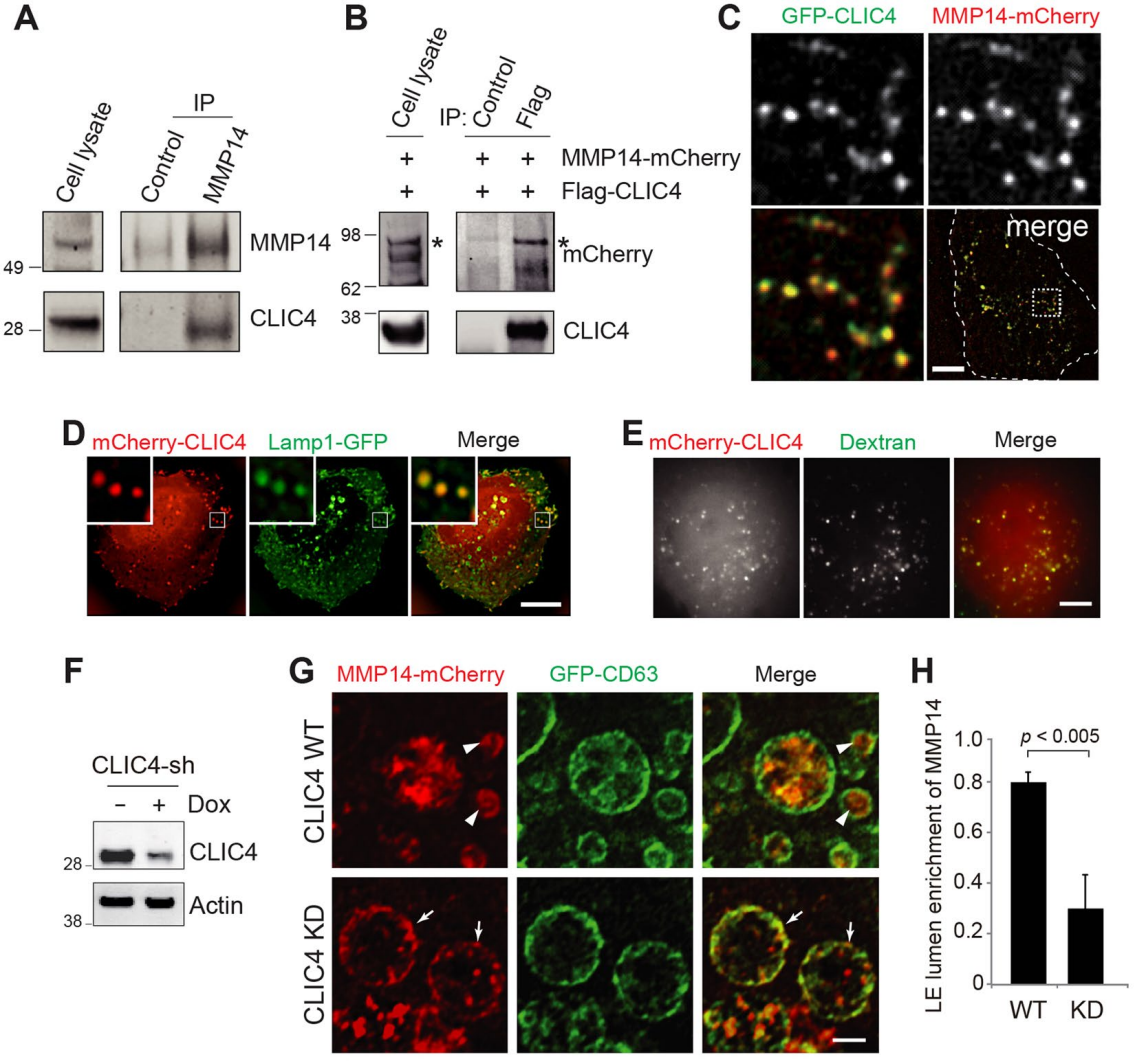
CLIC4 interacts with MMP14 and is required for LE luminal expression of MMP14. (**A**) ARPE19 cell lysates and the immunoprecipitants pulled down by anti-MMP14 or control mouse antibody were analyzed by immunoblotting with the indicated rabbit antibodies. (**B**) The lysates of 293 T cells transfected with Flag-CLIC4 and MMP14-mCherry (left) and the immunoprecipitants pulled down by anti-Flag or control antibody (right) were immunoblotted with the indicated antibodies. Asterisks point to the MMP14-mCherry specifically co-immunoprecipitated with CLIC4. (**C**) Live snapshot image of co-transfected GFP-CLIC4 and MMP14-mCherry in ARPE19 cells. The low magnification photograph of a single cell is shown in the bottom right panel; the dashed lines mark the cell border. The boxed area is magnified to highlight the overlapping GFP-CLIC4 and MMP14-mCherry signals. (**D**) A still image of live ARPE19 cells transfected with mCherry-CLIC4 (red) and Lamp1-GFP (green). The low-magnification images taken from a single cell and the enlarged views of the boxed area (insets) are shown. (**E**) A snapshot of live images shown in Supplementary Movie 1. Fluorescein-dextran was added to the culture medium of ARPE19 cells transiently transfected with mCherry-CLIC4. After washing and a 6-hour chase, the cells were imaged in recording buffer. (**F**) Immunoblots of Dox treated (+) or untreated (−) ARPE19 cells stably expressing inducible CLIC4-sh1 plasmid (no fluorescent reporter) probed with the indicated antibodies are shown. (**G**) The ARPE19 cell line described in (**F**) was treated with (i.e., CLIC4 KD) or without (CLIC4 WT) Dox for 3 days. These cells were then transfected with MMP14-mCherry, GFP-CD63, and Flag-Rab5 Q79L for 1 day in the same Dox conditions before fixation and staining. Representative super-resolution confocal images of mCherry and GFP stained cells are shown. Arrowheads and arrows point to LE luminal and LE limiting membrane signals of MMP14, respectively. (**H**) Quantification of targeting of MMP14 in (**G**). For quantification, a threshold was adjusted to exclusively reveal the limiting membrane signal of CD63. In 120 randomly selected LEs, the ratio of the MMP14 signal within the LE lumens was deducted by subtracting its intensity on limiting membrane from total intensity on the entire LE globe. n = 120 endosomes from 24 cells. Scale bars (in **C**–**E**,**G**) = 10 µm.

Given that both CLIC4 and MMP14 were enriched in the CD63-expressing LE fractions (Fig. 4D), we tested the plausible physical interaction between CLIC4 and MMP14. We showed that the anti-MMP14 antibody, but not the isotype-matched control antibody, specifically co-immunoprecipitated CLIC4 from the detergent lysates of either ARPE19 cells (Fig. 5A) or mouse retinas (Fig. S2A). The reverse immunoprecipitation showed that anti-CLIC4 antibody also specifically pulled down MMP14 as well (Fig. S2B). Furthermore, the 293T cell transfected MMP14-mCherry was specifically co-immunoprecipitated by Flag-CLIC4 using the anti-Flag antibody (Fig. 5B).

The live cell imaging showed that the ectopically expressed GFP-CLIC4 and MMP14-mCherry were extensively colocalized in LE-like granular structures in ARPE19 cells (Fig. 5C; Pearson’s correlation coefficient was 0.71 ± 0.14, n = 5 cells). To demonstrate the LE residence of CLIC4, we showed that in live ARPE19 cells, the granular mCherry-CLIC4 signal was extensively colocalized with the LE highlighted by either the transfected Lamp1-GFP (Fig. 5D) or extracellularly loaded FITC-dextran (Fig. 5E, Movie 1).

### CLIC4 is important for the LE luminal expression of MMP14

We used super-resolution confocal microscopy to examine whether the expression level of CLIC4 affects the LE expression of MMP14. In these experiments, the transfected GFP-CD63 was used to highlight the LE, and co-transfected Rab5(Q79L) was used to expand the size of the LE to increase the visibility of its lumen contents^57^. The ARPE19 cells stably expressing a Dox-inducible CLIC4-sh plasmid (that did not encode the fluorescent protein reporter) were used (Fig. 5F). In the “WT” cells (i.e., without Dox treatment), MMP14-mCherry was predominantly localized in the lumen of the LE (arrowheads, Fig. 5G). In contrast, when CLIC4 was suppressed by Dox treatment, the LE expressed MMP14-mCherry was preferentially found on the limiting membrane instead (arrows, Fig. 5G). The mislocalized MMP14-mCherry between the LE vacuoles were also increased the CLIC4 KD cells. The quantification assays showed that the reduced LE luminal enrichment of MMP14-mCherry in CLIC4-KD cells was statistically significant (Fig. 5H). These results suggest that CLIC4 modulates the sorting of MMP14 into the LE lumens.

### CLIC4’s late domain participates the LE lumen sorting of MMP14

**Figure 6:**
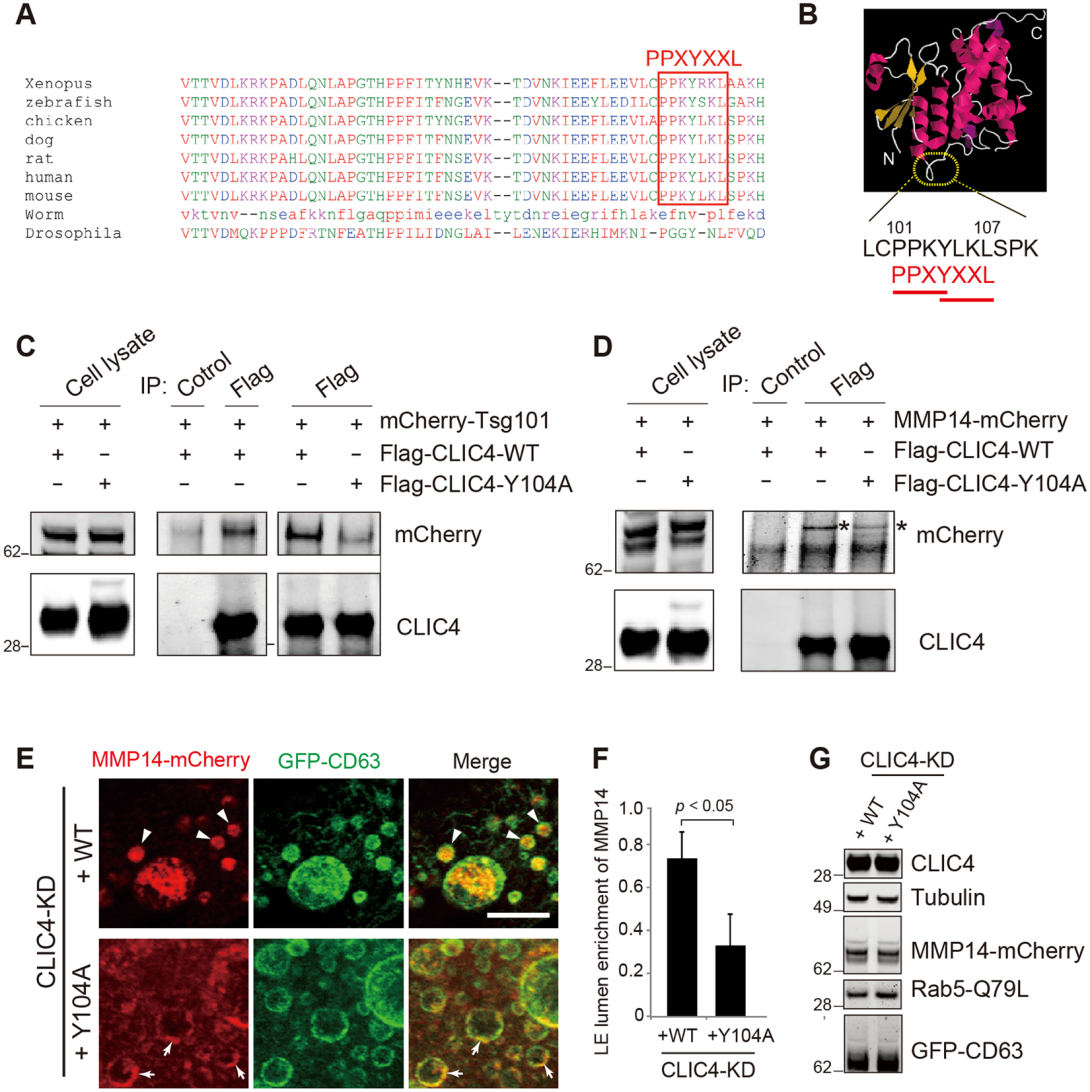
Late domains of CLIC4 modulate the LE lumen sorting step of MMP14 trafficking. (**A**) Amino acid alignments of CLIC4 from different species. The two overlapping late domains (PPXY and YXXL) are highly conserved in vertebrates, but less conserved in invertebrates. (**B**) The putative dual late domain sequence in human CLIC4. The CLIC4 protein structure was imaged (Protein Data Bank ID: 2AHE. Doi: 10.2100/pdb2ahe/pdb) using online JSmol software. (**C**) (Left panels) The representative immunoblots of total lysates of 293 T cells transfected with mCherry-Tsg101 together with Flag-CLIC4-WT or Flag-CLIC4-Y104A and probed by the indicated antibodies. (Middle panel) The immunoblots of the immunoprecipitants pulled down by the control or anti-Flag antibody from the indicated transfected cell lysates. (Right panel) Immunoblots of Flag immunoprecipitants isolated from the indicated transfected cell lysates. Compared to its WT counterpart, the Flag-CLIC4-Y104A pulled down less mCherry-Tsg101. (**D**) The total lysates (left panels) as well as the immunoprecipitants pulled down by anti-Flag or control antibody (right panels) were immunoblotted by the indicated antibodies. Asterisks point to the MMP14-mCherry species that was specifically co-immunoprecipitated with Flag-CLIC4. (**E**) ARPE19 cell line expressing inducible CLIC4-sh was treated with Dox for 3 days and then transfected with MMP14-mCherry, GFP-CD63, together with CLIC4-WT-IRES-FLAG-Rab5 Q79L, or CLIC4-Y104A-IRES-FLAG-Rab5 for 1 day in the same Dox conditions. Representative confocal images of mCherry and GFP stained cells are shown. Arrowheads and arrows point to LE luminal and LE limiting membrane signals of MMP14, respectively. (**F**) MMP14 intensity ratio in LE lumen for (**E**). n = 120 endosomes from 24 cells. Bars show means ± S.D. from three independent experiments. p value, t-test. Scale bar = 10 µm. (**G**) Immunoblots demonstrate the same amount of ectopically expressed proteins in the rescue experiments (in **E**,**F**).

CLIC4 exhibits an N-terminal thioredoxin-like domain and an α-helical C-terminal domain and its structure is related to the omega-class glutathione S-transferases^58^. In the flexible loop region between these two domains (amino acids 101–107), we noted two overlapping conserved late domain motifs (i.e., PPXY and YXXL) (Fig. 6A,B). The late domain found in several membrane-enveloped viral proteins (e.g., HIV Gag) is known to bind to Tsg101^59^. Tsg101 is a component of the endosomal sorting complex required for transport (ESCRT) complex known to recruit and sort membrane cargoes into the lumen of LEs^23,60^. Thus, we asked whether CLIC4 also binds to Tsg101 through its late domain. Using co-immunoprecipitation, we showed that anti-Flag antibody specifically pulled down mCherry-Tsg101 in 293 T cells transfected Flag-CLIC4, (Fig. 6C, middle panel). The Flag-CLIC4 variant Y104A, in which the conserved tyrosine in the late domains was altered to alanine, had 40.44 ± 17.6% (p = 0.028; n = 3) reduced ability to bind to mCherry-Tsg101. Interestingly, CLIC4-Y104A also showed 56 ± 15.7% (p = 0.041; n = 3) less ability to co-immunoprecipitate with MMP14-mCherry (Fig. 6D), indicating that the late domain is a motif involved in multiple protein-protein interactions.

Finally, we performed rescue experiments by transfecting dog CLIC4 cDNAs into ARPE19 lines expressing human-specific CLIC4-sh. We showed that when WT CLIC4 was reintroduced, MMP14-mCherry was predominantly localized in the LE lumen (Fig. 6E). These results excluded the off-target effect exerted by CLIC4-sh and showed the specific requirement of CLIC4 for the LE targeting of MMP14. In contrast, reintroducing CLIC4 Y104A into the CLIC4-KD RPE cells did not restore the LE lumen localization of MMP14 (Fig. 6E,F). We used the immunoblotting assays to confirm that the level of both re-introduced CLIC4 variants (and other transfected proteins) in the rescue experiments was similar (Fig. 6G). These results collectively support the notion that the late domain of CLIC4 is important for the LE luminal sorting of MMP14.

### CLIC4 suppression inhibits the apical secretion of MMP2 in polarized RPE monolayers

**Figure 7:**
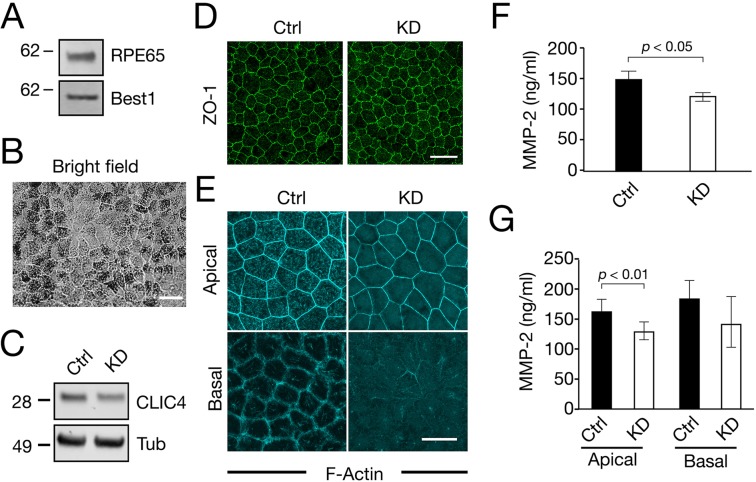
CLIC4 modulates the apical secretion of MMP2 in polarized human RPE monolayers. (**A**,**B**) RPE monolayers differentiated from the human embryonic cell line stably expressing Tet-regulated CLIC4-shRNA were analyzed by immunoblotting assays using the indicated antibodies (**A**; Best1: Bestrophin1) and by bright-field microscopy (**B**). (**C**–**G**) The above described human RPE monolayers, treated without Dox (Ctrl) or with Dox (KD) for 5–9 days, were analyzed by immunoblotting assays (C; Tub: β-tubulin), ZO-1 staining (**D**), F-actin staining (**E**), and MMP2 secretion (**F**,**G**). In (**E**), the confocal images sectioned through the apical and basal sides of the cells are shown. The concentration of the MMP2 was obtained from the cells grown on 38 mm^2^ (**E**), and 11 mm^2^ (**F**) surface areas, respectively. Bars show means ± S.D. N = 3. p, t-test. Scale bars = 30 µm (**B**,**D**); 20 µm (**E**).

Compared to ARPE19 cells, polarized RPE monolayers better mimic RPE tissue *in situ*. However, the long period of time required for the cells to polarize makes this cell model incompatible with our cell-based assays, in which the degradation foci formed within a couple of hours after cell plating. Furthermore, our several attempts showed that primary RPE cells dissociated from mouse retinas failed to adhere to the gelatin within the assay time frame. Therefore, to assess CLIC4’s role in MMP14 activation in polarized RPE cells, we measured secreted MMP2 as a proxy. To this end, we generated a human embryonic stem cell line stably expressing Tet-regulated CLIC4-shRNA. These cells were first differentiated into RPE cells in the absence of Dox. The RPE fate differentiation of these cells was confirmed by the expression of two RPE markers (RPE65, Bestrophin1) (Fig. 7A) and pigment granules (Fig. 7B). Furthermore, the human RPE monolayers formed ZO-1 labeled tight junctions (Fig. 7D), and F-actin labeled apical microvilli and basal membrane specializations (Fig. 7E). The latter confirmed the RPE monolayers were properly polarized.

We suppressed CLIC4 in the polarized RPE monolayers by adding Dox 5–9 days before the assays. The KD of CLIC4 was confirmed by immunoblotting assays (Fig. 7C; 53 ± 14% relative to the cells without treating Dox, N = 3). While the CLIC4-KD cells had typical looking tight junctions (Fig. 7D), these cells had apparent loss of the apical microvilli and basal membrane specializations (Fig. 7E).

Using the Multiplex enzyme-linked immunosorbent assay (ELISA) previously described^47^, we measured the concentration of the MMP2 secreted from the RPE monolayers containing the same number of the cells. When the monolayers were grown in the culture plates, significantly lower concentration of total MMP2 was detected in the cultured media of CLIC4-KD cells compared to the control cells. Interestingly, when the monolayers were grown on Transwell filters, the secretion of MMP2 from the apical, but not the basal, side of the cells was selectively reduced by CLIC4 silencing (Fig. 7G). In contrast, the secretion of MMP1 and MMP9 was not significantly altered by CLIC4 KD (Fig. S4).

## Discussion

This paper makes several advances in our understanding of ECM remodeling and the endosomal trafficking of RPE cells. (i) Here we investigated the dynamic pericellular gelatin degradation function of ARPE19 cells. These studies complemented the previous reports which used zymography for the secreted gelatinase activity^42–44^ and the structural characterization of the ECM formed underneath the long-term ARPE19 cultures^46^. (ii) Our studies show that in ARPE19 cells MMP14 is central to the genesis of the degradation foci at the focal adhesions. While we did not test it directly, the locally produced MMP2 might further contribute, in part, to the formation and/or the size of the gelatin degradation foci. (iii) Mechanistically, we showed that CLIC4 modulates the matrix degradation activity of MMP14, through the recruitment of MMP14 onto the lipid rafts and LE lumens in ARPE19 cells. (iv) CLIC4-KD suppressed the secretion of MMP2 from the apical side of the polarized RPE cells.

### CLIC4 regulated MMP14 matrix degradation activity

While the pathway that underlies the surface internalization and surface recycling of LE-expressed MMP14 has been investigated^21,22,26,61^, little is known about the LE targeting of MMP14. The current study shows that the LE sorting of MMP14 importantly regulates its ECM degradation activity. CLIC4 silencing concomitantly reduces the expression of MMP14 in the LE lumen and the lipid rafts, the autoproteolytic cleavage of MMP14, and the focal dissolution of ECM in ARPE19 cells. Possibly due to the transient nature of its membrane insertion^58,62^, CLIC4 itself was not readily detected in the lipid rafts of the ARPE19 cells.

Lipid raft-rich and acidic microenvironments favor biased peptidyl cleavage^63^. Coincidently, both the LE luminal membranes and the focal adhesions have abundant lipid rafts^64–67^. The LE lumen and certain MMP-activated pericellular regions are also known to have a low pH milieu^68–70^. Thus, we propose that MMP14-expressed on LE luminal membranes contributes to the formation of the MMP14-active microdomains in the degradative focal adhesions. In this model, the LE is used for not only storing the intracellular MMP14 but also regulating its matrix proteolytic activity. In line with this concept, perturbing the key LE trafficking regulator Rab7 impairs MMP14-mediated activity for cancer cell migration and invasion^20^.

### CLIC4 is a novel LE targeting modulator

This manuscript reveals CLIC4 as an important modulator that interacts with the ESCRT complex for LE cargo sorting. In the LEs, the ESCRT-0 complex components recruit ubiquinated cargoes, while ESCRT-I and ESCRT-II coordinate the limiting membrane invagination, and ESCRT-III pinches off membranes into the LE lumens. MMP14 is known to be ubiquitinated by Nedd4, which binds to the ESCRT-1 component Tsg101^71,72^. Tsg101 and MMP14 both bind to CLIC4 (this study). This protein network could explain the sequestering of ubiquinated MMP14 into the LE lumen. Like MMP14, the LE expression of CD63 also appeared to be reduced in CLIC4-KD ARPE19 cells (Fig. 5G). Plausibly, CLIC4 acts as a novel regulator involving the sorting of multiple LE components. Further studies will be required to verify this claim. Emerging evidence shows that the late endocytic compartments participate in a wide array of cellular events (e.g., protein sorting, lipid homeostasis, and metabolite signaling^73^). The role(s) played by CLIC4 in these LE functions of RPE cells warrants further investigation.

Another novelty of this paper is that we showed that CLIC4 contains a functional late domain. The late domain, which was initially identified in viral proteins, is known to critically participate in the late stage of virus budding^59,74^. Only two late domain-containing human proteins, syntenin and the G protein-coupled receptor Par1, have so far been reported^75,76^. Syntenin and Par1 both bind to the ESCRT-III complex component and mediate the luminal vesicle budding and cargo sorting of the LE, respectively. CLIC4 has two different classes of late domains (PPXY, YXXL) that are arranged in tandem and overlap at the Y104 residue. Using the site-directed mutant Y104A, we showed that the late domains of CLIC4 are required for the LE lumen expression of MMP14. The overlapping late domains have been proposed to serve as a mechanism for redundancy and/or interdependence of weak protein-protein interactions^74^. We found that the Y104A mutant of CLIC4 has reduced binding ability to both Tsg101 and MMP14. Whether the interaction between CLIC4, Tsg101, and MMP14 is direct or not awaits further investigation.

It is known that when LEs fuse to the plasma membrane, the intraluminal LE cargoes are released into the extracellular space in the exosomes. Both MMP14 and Tsg101 have been previously detected in the exosomes secreted by several cell types^21,77^. We confirmed the expression of MMP14, Tsg101, and CLIC4 in exosomes purified from the ARPE19 culture media (Fig. S3). The exosomal expression of MMP14 had no detectable change in CLIC4 KD cells cultured in dishes (Fig. S3). It will be of future interest to test whether CLIC4 selectively regulates the exosome secretion of MMP14 from the apical or basolateral side of the cells.

As mentioned, CLIC3 regulates the retrieval of LE-expressed MMP14 back to the degradative invadopodia in MDA-MB-231 cancer cells^21,22,26^. Our finding about CLIC4 adds additional support to the growing body of evidence that several CLICs have important trafficking roles in endocytic-phagocytic pathways^28,49,78^, in addition its proposed role as a channel. The observations also suggest that distinct CLIC members might play distinct but parallel roles in transporting cargoes to different organelles or in different cell types.

### CLIC4 regulates the genesis of matrix degradative focal adhesions in cultured RPE cells

While invadopodia have long been considered to be matrix degradation organelles, only recently, focal adhesions were shown to be bona fide matrix degradation organelles in some cancer cell lines^18,79^. Here we showed that in ARPE19 cells, most gelatin degradation occurs robustly in the proximity of focal adhesions. Although CLIC4 is essential for transforming the canonical focal adhesions into the degradative organelle, it is dispensable for focal adhesion assembly per se in ARPE19 cells. Previous studies showed that CLIC4-suppressed HeLa cells formed fewer and smaller focal adhesions on collagen or fibronectin and had reduced cell adhesion^49^. The difference between these two reports with regards to CLIC4’s role in focal adhesion formation might be attributed to the differences in the cell type and/or substratum. Also, while in the present study we conditionally suppressed CLIC4, in the previous HeLa cell study CLIC4 was chronically suppressed. Prolonged CLIC4 suppression could conceivably generate the negative feedback, leading to the compromised focal adhesion assembly.

### Implications of CLIC4-regulated MMP14 activation in normal and pathogenic RPE cells

Since the gene expression, morphology, and several other cellular properties of ARPE19 cells are distinct from RPE monolayers of normal retinas *in vivo*^80^, the MMP14-localized degradation foci in the RPE cells *in situ* remains to be shown. In fact, the structure of the focal adhesion has thus far been defined and well-studied in subconfluent 2D cell cultures. The structural and functional equivalent(s) of focal adhesions are, however, unclear in 3D cultures and *in vivo*. Nevertheless, it is interesting to note that several focal adhesion components, including integrin alpha(v) beta5 (the key receptor for the photoreceptor outer segment engulfment) and focal adhesion kinase, are all predominantly distributed in the apical microvillar surface of the RPE *in situ*^38,81,82^. The RPE microvilli and the photoreceptor outer segments are co-embedded in the ECM-rich microenvironment known as the interphotoreceptor matrix. Since RPE cells perform daily engulfment of photoreceptor outer segments, the interphotoreceptor matrix must undergo constant remodeling as part of tissue repair^83^. We speculate that a yet-to-be identified cytostructure that dynamically forms in the RPE microvillar membranes represents the functional analogue of degradative foci, and that this particular structure contains the focal adhesion components and MMP14. This spatially regulated activation of the MMP14 in RPE cells might be central to maintaining the normal homeostasis of the retina-RPE-choroid complex.

Previous studies showed that the amount of secreted MMP2 in the cultured media of polarized human RPE monolayers is directly proportional to its gelatinase activity; the pro-MMP2 was trapped in the ECM instead^47^. The finding that CLIC4 KD inhibits the apical secretion of MMP2 suggests that CLIC4 regulates the ECM remodeling in polarized RPE cells, possibly by modulating the MMP14 activity in the apical surfaces. We ruled out the possibility that reduced MMP2 secretion was due to the decreased transcription of MMP2 or MMP14 based on our unpublished RNAseq data. Nonetheless, since MMP14 mediated MMP2 activation also requires TIMP-2^84^, the details of the molecular mechanism that underlie the CLIC4-regulated MMP2 secretion remain to be further explored.

Several MMPs have been linked with the pathogenesis of proliferative vitreoretinopathy and AMD^39,83,82^. Altered MMP14 expression has been found in AMD animal models^42,85,86^, and the genetic variants of MMP2 and TIMP-3 (an MMP14 inhibitor) confer the risk of AMD progression^87^. In both proliferative vitreoretinopathy and AMD, RPE cells undergo dedifferentiation (or transdifferentiation) in conjunction with dysregulated ECM remodeling^88,89^. In that sense, investigating the dynamic matrix proteolysis of ARPE19 cells may provide useful insights into the early-stage pathogenesis of these eye diseases. Finally, soft drusens, which formed between the (basal) RPE and Bruch’s membrane, as well as subretinal drusenoid deposits, which formed between the (apical) RPE and neural retinas, are clinical features that confer the risk for end-stages of RPE atrophy in AMD^90,91^. The primary causes that underlie the deposition of the irregular ECM by the atrophic RPE cells and the relationship between these spatially distinct retinal lesions remain unknown. Future investigation of the CLIC4-reguated MMP14 activation *in vivo* is an important subject for understanding its physiological relevance to retinal histopathology.

## Methods

### Reagents

Antibodies used in this study included actin (mouse, Santa Cruz, sc-8432, 1:250), CD63 (mouse H5C6, DSHB, 1:1000), mCherry (goat, Sicgen, AB0040-200, 1:1000), CLIC4 (rabbit, Millipore ABS2088;1:500), Flag (mouse Sigma F3165, 1:2000), MMP14 (rabbit, Abcam ab51074, 1:500 for IFA, 1:1000 for WB; epitope: amino acid ~150–180); MMP14 (mouse, Santa Cruz sc-373908, 2 µg for immunoprecipitation; epitope: amino acids 431–462), vinculin (mouse, Sigma V9131, 1:500), flotillin-1 (mouse, BD Transduction Laboratories, Clone 18, 1:1,000), GAPDH (rabbit, Cell Signaling Technology, 14C10, 1:5000), Tsg101 (goat, Santa Cruz M-19, 1:100), Bestrophin1 (rabbit; Proteintech 21910-1-AP, 1: 1,000), RPE65 (rabbit, gift of Jian-Xing Ma, 1:1000), β-tubulin (mouse, Sigma-Aldrich Cat# T5201, 1:2,000), ZO-1 antibody (rabbit Zymed 61–7300 or mouse Zymed 33–9100, 1:500), Alexa-dye conjugated secondary antibodies (made in donkey, Thermo Fisher Scientific, 1:400), Alexa-dye conjugated phalloidin (Thermo Fisher Scientific, 1:1000), and IRDye-conjugated secondary antibodies (LI-COR; 1:10,000).

MMP14-mCherry (a gift of Dr. Philippe Chavrier)^25^, GFP-CD63 (a gift of Francisco Sánchez-Madrid^92^, mCherry-Tsg101 (Addgene # 38318^93^), dog CLIC4^28^ and Flag-Rab5(Q79L)^56^ were previously described. All constructs were generated using standard cloning techniques. The late domain mutation (Y104A) was introduced by site-directed mutagenesis in dog CLIC4 and confirmed by sequencing. The MMP14-sh1 targeting sequence, 5′-GGGTGAGGAATAACCAAGTGAT-3′, was inserted downstream of the U6 promoter followed by a CAG-IRES-mCherry cassette. The MMP14-sh2 targeting sequence, 5′-TGGCAAATTCGTCTTCTTCAA-3′, was cloned into the TRIPZ vector wherein the shRNA and turboRFP could be co-induced upon Dox induction. TRIPZ-lenti-turboRFP-CLIC4-sh1 (5′-GTCATTTCATTGCCATCCA-3′) and turboRFP-CLIC4-sh2 (5′-TATCAACTAGAATAGAGAG-3′) were from Thermo Fisher Scientific. For the non-colored version, the turboRFP coding sequence was deleted from the above constructs. The TRIPZ-lenti plasmid was co-transfected with psPAX2 packaging plasmid, and the pMD2-G envelope plasmid in 293 T cells to produce lentivirus. The resulting medium supernatant containing lentiviral particles was filtered, spun, and precipitated with the Lenti-X Concentrator (Clontech).

### Cell lines, cell transfection, viral infection, RPE differentiation of human ES cells

All tissue cultures reagents were obtained from Thermo Fisher Scientific unless otherwise mentioned. The breast cancer cell line MDA-MB-231 (gift of Dr. Yi Li) was maintained in DMEM (Cellgro) with 10% FBS. ARPE19 cells (obtained from ATCC) were maintained in MEMα medium (Sigma) supplemented with 5% fetal bovine serum, Glutamine, N1 supplement, non-essential amino acids, Taurine, Hydrocortisone, and Triiodo-thyronin^94^. Either Amaxa® electroporation (Lonza) or Lipofectamine were used to transfect ARPE19 cells. ARPE19 Tet-on inducible stable lines were generated by lentivirus infection followed by puromycin selection. These stable lines were maintained in the medium with Tet-free fetal bovine serum (Clontech). Dox (0.4 µg/ml) was applied for 3 days to induce gene silencing before the assays.

Human ES H1 cell line was purchased from WiCell and maintained in mTeSR-1 medium (STEMCELL Technologies). All human ES cell work has been approved by Weill Cornell ESCRO. The H1 cells were transduced by the CLIC4-sh lentivirus (with 4 μg/ml polybrene) followed by puromycin (0.5 μg/ml) selection. The H1 Tet-on inducible stable line was then differentiated into RPE with the previously published protocol^95^. Briefly, the cells were cultured to confluence in 6-well culture dishes pretreated with 1:50 diluted Matrigel (Corning) in differentiation medium consisting of Knock-Out (KO) DMEM, 15% KO serum replacement, 1% nonessential amino acids, 2 mM glutamine, 50 U/ml penicillin-streptomycin, and 10 mM nicotinamide (Sigma-Aldrich, #N0636) for the first 14 days. 100 ng/ml human Activin-A (PeproTech, #120–14) was added into differentiation medium for the culture between the 15^th^–28^th^ days of differentiation and then removed until differentiation was completed. About 8–10 weeks later, we manually picked the pigmented clusters and, then plated on Matrigel-coated dishes or Transwell filters in RPE culture medium^94^. RPE cells were plated in 12-well plates or 12-well Transwell filters (Corning) at a density of 1 × 10^5^ cells/cm^2^ for another 6–8 weeks to allow them to form monolayers. Afterward, CLIC4 expression was suppressed by adding Dox (0.4 µg/ml) for an additional 5–9 days. The conditioned media were collected for MMP secretion assays, and the cells were either fixed for immunostaining or harvested for immunoblotting. All the RPE cells used in this study were at passage 1 (i.e., no passage).

### MMP secretion assay

The conditioned media were collected from human RPE monolayers cultured in 12-well plates or 12-well Transwells. The media were spun (1,500 rpm 5 min) and then subjected to Multiplex sandwich ELISA using the Bio-Plex 200 System (Bio-Rad). Human MMP magnetic Bead Panel 2 kit (MMP-1, 2, 7, 9 and 10) was used according to the manufacturer’s instruction (Millipore Sigma). Standard curves for each MMP were generated by using the reference concentrations supplied by the manufacturers. Raw data (mean fluorescent intensity) from all kits were analyzed by Bio-Plex Manager Software (Bio-Rad) to obtain concentration values. Each multiplex assay was performed in duplicate, and three independent samples were tested. Samples were normalized to cell numbers.

### Fluorescein gelatin degradation assay

Gelatin degradation assays were carried out as previously described with minor modifications^96^. Briefly, 12-mm glass coverslips were crosslinked with 0.5% glutaraldehyde and then coated with 0.05% fluorescein-conjugated gelatin (Thermo Fisher Scientific) in 2% sucrose-containing phosphate buffered saline (PBS). The coverslips were then quenched with 50 mM NH_4_Cl/PBS for 10 min, washed, and sterilized with ethanol. In some experiments, cells were pre-treated with GM6001 (100 nM for 5 hours; Sigma) or DMSO (0.1%) before plating. The chemicals were also added to the cells during plating. In some experiments, a mixture of equal amounts of inducible CLIC4-KD ARPE19 stable lines, previously treated with or without Dox for 3 days, were plated together (1 × 10^4^ cells each population/0.4 ml medium/12 mm coverslip). In some experiments, ARPE19 cells were transiently transfected with MMP14-mCherry, mCherry, or MMP14-sh (for the time periods as indicated in the legends) before plating. For all gelatin degradation assays, cells were harvested 5–6 hours after plating by 4% paraformaldehyde fixation. The coverslips were directly subjected to wide field fluorescence microscope imaging (Axio Observer.Z1; Zeiss). Images taken under the same acquisition conditions (20x lens) were subjected to ImageJ quantification analysis. A cell that possessed at least 10 clear dark loci within the cell or at the cell periphery was defined as a positive cell for quantification.

### Immunostaining, dextran uptake assay, and imaging analysis

For immunostaining, cells were fixed with 4% paraformaldehyde for 10 min and quenched with 50 mM NH_4_Cl for 10 min, followed by 1 hour in blocking buffer PBTAD (0.5% BSA, 0.2 mg/ml sodium azide, 0.25% Triton X-100, and 4′,6-diamidino-2-phenylindole (DAPI)). The samples were then incubated with primary antibodies in the same buffer for 1 h and then secondary antibodies for 30 min. After three washes, the stained cells were mounted with ProLong Gold Antifade reagent (Thermo Fisher Scientific). For surface MMP14 labeling, cells were fixed with 2% paraformaldehyde for 5 min, and then stained with buffer containing no detergent during the entire procedure.

All images were acquired by either a Leica TCS SP2 spectral confocal system (63x objective, 0.3 µm thick) or LSM880 confocal microscope and 63x oil immersion lens (Carl Zeiss). For the LE lumen localization study of MMP14, super-resolution grade confocal scanning (Zeiss LSM880+ airyscan head mode) was used. For live imaging experiments, cells were grown in glass-bottom dishes (Greiner Bio-One) and placed in live cell imaging solution (Hank’s balanced salt solution supplemented with 1% FBS and 1.0 g/L glucose). The imaging was carried out by a wide field fluorescence microscope (Axio Observer.Z1; Carl Zeiss), equipped with either the Plan-Apochromat 63×/1.4 oil immersion objective, the AxioCam HRM camera, the CO_2_ Module S, and the TempModule S (Zeiss) or Zeiss LSM880 with incubation chamber. Intensity and area of the vinculin-positive focal adhesions as well as the surface MMP14, were quantified using ImageJ software. For dextran uptake assay, the cells were fed with 1 mg/ml of fluorescein-conjugated dextran (10,000 MW, Thermo Fisher Scientific) for 24 hours, and incubated with the medium without dextran for an additional 6 hours, then followed by live imaging as described above. For presentation purposes, some images were processed with a deconvolution plugin (Deconvolution Lab) in ImageJ using the Tikhonov-Miller algorithm. The level of colocalization of two proteins (i.e., Pearson’s correlation coefficient) was analyzed by using the Colocalization Index plugin in ImageJ.

### Immunoprecipitation and immunoblotting assays

CD1 mouse retinas, ARPE19 cells or the transfected 293 T cells were lysed with RIPA buffer (50 mM Tris pH 7.4, 150 mM NaCl, 0.5% NP-40, 0.5% sodium deoxycholate, 1 mM EGTA, 5% glycerol) plus 0.5% 3-((3-cholamidopropyl) dimethylammonio)-1-propanesulfonate (CHAPS), 1 mM phenylmethylsulfonyl fluoride (PMSF), and cocktails of proteinase inhibitor and phosphatase inhibitor. The lysates were precleared with Protein-G Dynabeads® (Thermo Fisher Scientific) at 4 °C for 1 hour. The binding reaction was carried out at 4 °C overnight. After 4 washes using the lysis buffer, the bound proteins were eluted with 1x Laemmli sample buffer. Immunoblotting assays were carried out using standard methods. Protein signals detected by primary antibody followed by IRDye-conjugated secondary antibodies were quantified with the Odyssey infrared image system (LI-COR). For the immunoprecipitation/immunoblotting experiments, inputs represent 5% of the total protein extracts used for immunoprecipitation. The quantification data were obtained from three independent repeats and the representative blots were shown.

### Surface biotinylation assay

ARPE19 cells plated on gelatin-coated dishes (10 cm diameter) for 5 hours were rinsed with ice-cold PBS, pH 8.0, and incubated with 2 mM Sulfo-NHS-LC-Biotin (Thermo Fisher Scientific) in PBS on ice for 30 min. Afterwards, the cells were quenched by three washes of 100 mM glycine in PBS before harvest for immunoprecipitation and immunoblotting assays. The biotinylated protein signal was detected by IRDye 800CW Streptavidin and quantified by Odyssey infrared image system.

### Sucrose gradient fractionation assay

The assay was carried out as described^97^ with minor modification. Briefly, ARPE19 cells suspended in ice-cold 10 mM Tris-Cl pH 7.4, buffer containing 0.25 M sucrose, 2 mM MgCl_2_, 1 mM EDTA, protease inhibitor cocktails, PMSF, and phosphatase inhibitors were homogenized by three passages through a ball-bearing homogenizer, and then centrifuged at 800 × g for 10 min at 4 °C. The post-nuclear supernatants were then centrifuged at 220,000 × g for 40 min at 4 °C. The pellets obtained were resuspended in 1% Triton X-100 containing PBS (plus protease inhibitor cocktails and phosphatase inhibitors) and then centrifuged at 90,000 × g for 10 min at 4 °C. The supernatant (membrane lysates) were loaded onto 2 ml linear gradients of 5–20% (w/w) sucrose solution containing PBS pH 7.4, 0.1% Triton X-100, and centrifuged at 220,000 × g centrifugation at 4 °C for 16 hours in a Beckman TLS-55 rotor. A total of 11 fractions (~100 μL) were collected from the bottom punctured by a 26-G needle. Equal volume (15μl) of each fraction was subjected to the electroporation followed by the immunoblotting assays.

### Detergent-resistant membrane analysis

ARPE19 stable lines, treated with or without Dox for 3 days, were passaged onto gelatin-coated dishes for 5 hours at 37 °C before harvest for the detergent-resistant membrane assays, as previously described^98^. The entire procedure was carried in 4 °C cold room. Briefly, the cells suspended in TNE buffer (150 mM NaCl, 2 mM EDTA, 50 mM Tris-HCl pH 7.4, protease inhibitors, PMSF, and phosphatase inhibitors) were sheared with a 25-G needle (25 strokes), and then lysed with Triton X-100 (final 1%). The lysate was adjusted to 40% (w/v) iodixanol by adding 60% iodixanol (OptiPrep; Sigma). About 600 µl of the mixture was transferred to a Beckman Ultra-Clear centrifuge tube, overlaid with 1.2 ml of 30% iodixanol (w/v) and then 0.2 ml of TNE buffer, followed by centrifugation at 55,000 rpm for 2 hours with a Beckman TLS55 rotor. 1 ml solution from the bottom was collected as the soluble fraction, and 1 ml from the top as the floating, detergent-resistant fraction. Data are presented as means ± S.D of three independent experiments, and statistically analyzed by Student’s t-test.

### Exosome purification and analysis

Exosomes were purified as previously reported^99^. Briefly, ARPE19 cells stably expressing inducible CLIC4-sh were cultured with the growth medium containing Dox (0.4 µg/ml) for 4 days. The cell cultures were then refreshed with exosome-depleted culture media, in the absence or presence of Dox, for an additional 4 days. The collected culture media were centrifuged at 500 × g for 10 min to remove the cell debris. To collect the exosomes, the supernatants were subjected to a stepwise centrifugation at 4 °C, including 3,000 × g for 20 min, 20,000 × g (Beckman SW 28 rotor) for 20 min, and 100,000 × g for 21 hours. The pellet was washed with PBS and centrifuge again by 100,000 × g (Beckman TLA-100.3 rotor) for 17 hours. The exosome-containing pellets were resuspended with PBS and subjected to Nanopartical tracking analysis (NanoSight). The average peak sizes of the extracellular vesicles secreted from the control and the CLIC4-KD cells are 103 and 105 nm, respectively. Equal amount of exosomal proteins was subjected to the immunoblotting assays.

## Supplementary information

Supplementary Materials

Movie 1

## References

[1] Bonnans C, Chou J, Werb Z (2014). Remodelling the extracellular matrix in development and disease. Nat Rev Mol Cell Biol.

[2] Barbolina MV, Stack MS (2008). Membrane type 1-matrix metalloproteinase: substrate diversity in pericellular proteolysis. Seminars in Cell & Developmental Biology.

[3] Itoh Y, Seiki M (2006). MT1-MMP: a potent modifier of pericellular microenvironment. J Cell Physiol.

[4] Yana I (2007). Crosstalk between neovessels and mural cells directs the site-specific expression of MT1-MMP to endothelial tip cells. J Cell Sci.

[5] Koziol A (2012). The protease MT1-MMP drives a combinatorial proteolytic program in activated endothelial cells. FASEB journal.

[6] Meyer TN (2004). Spatiotemporal regulation of morphogenetic molecules during *in vitro* branching of the isolated ureteric bud: toward a model of branching through budding in the developing kidney. Dev Biol.

[7] Weaver SA (2014). Basal localization of MT1-MMP is essential for epithelial cell morphogenesis in 3D collagen matrix. J Cell Sci.

[8] Mori H (2013). Transmembrane/cytoplasmic, rather than catalytic, domains of Mmp14 signal to MAPK activation and mammary branching morphogenesis via binding to integrin beta1. Development.

[9] Toth M (2005). Cleavage at the stem region releases an active ectodomain of the membrane type 1 matrix metalloproteinase. Biochem J.

[10] Nakahara H (1997). Transmembrane/cytoplasmic domain-mediated membrane type 1-matrix metalloprotease docking to invadopodia is required for cell invasion. Proc Natl Acad Sci USA.

[11] Li H (1998). Immunological characterization of cell-surface and soluble forms of membrane type 1 matrix metalloproteinase in human breast cancer cells and in fibroblasts. Mol Carcinog.

[12] Galvez BG, Matias-Roman S, Albar JP, Sanchez-Madrid F, Arroyo AG (2001). Membrane type 1-matrix metalloproteinase is activated during migration of human endothelial cells and modulates endothelial motility and matrix remodeling. J Biol Chem.

[13] Zucker S (1998). Tissue inhibitor of metalloproteinase-2 (TIMP-2) binds to the catalytic domain of the cell surface receptor, membrane type 1-matrix metalloproteinase 1 (MT1-MMP). J Biol Chem.

[14] Poincloux R, Lizarraga F, Chavrier P (2009). Matrix invasion by tumour cells: a focus on MT1-MMP trafficking to invadopodia. J Cell Sci.

[15] Monteiro P (2013). Endosomal WASH and exocyst complexes control exocytosis of MT1-MMP at invadopodia. J Cell Biol.

[16] Takino T, Saeki H, Miyamori H, Kudo T, Sato H (2007). Inhibition of membrane-type 1 matrix metalloproteinase at cell-matrix adhesions. Cancer Res.

[17] Takino T (2006). Membrane-type 1 matrix metalloproteinase modulates focal adhesion stability and cell migration. Exp Cell Res.

[18] Wang Y, McNiven MA (2012). Invasive matrix degradation at focal adhesions occurs via protease recruitment by a FAK-p130Cas complex. J Cell Biol.

[19] Sato H (1994). A matrix metalloproteinase expressed on the surface of invasive tumour cells. Nature.

[20] Williams KC, Coppolino MG (2011). Phosphorylation of membrane type 1-matrix metalloproteinase (MT1-MMP) and its vesicle-associated membrane protein 7 (VAMP7)-dependent trafficking facilitate cell invasion and migration. J Biol Chem.

[21] Hakulinen J, Sankkila L, Sugiyama N, Lehti K, Keski-Oja J (2008). Secretion of active membrane type 1 matrix metalloproteinase (MMP14) into extracellular space in microvesicular exosomes. Journal of cellular biochemistry.

[22] Loskutov YV (2015). NEDD9/Arf6-dependent endocytic trafficking of matrix metalloproteinase 14: a novel mechanism for blocking mesenchymal cell invasion and metastasis of breast cancer. Oncogene.

[23] Woodman PG, Futter CE (2008). Multivesicular bodies: co-ordinated progression to maturity. Curr Opin Cell Biol.

[24] Piper RC, Katzmann DJ (2007). Biogenesis and function of multivesicular bodies. Annu Rev Cell Dev Biol.

[25] Rosse C (2014). Control of MT1-MMP transport by atypical PKC during breast-cancer progression. Proc Natl Acad Sci USA.

[26] Macpherson IR (2014). CLIC3 controls recycling of late endosomal MT1-MMP and dictates invasion and metastasis in breast cancer. J Cell Sci.

[27] Ashley RH (2003). Challenging accepted ion channel biology: p64 and the CLIC family of putative intracellular anion channel proteins (Review). Molecular Membrane Biology.

[28] Chou SY (2016). CLIC4 regulates apical exocytosis and renal tube luminogenesis through retromer- and actin-mediated endocytic trafficking. Nat Commun.

[29] Riggins KS (2010). MT1-MMP-mediated basement membrane remodeling modulates renal development. Exp Cell Res.

[30] Daruich A (2015). Central serous chorioretinopathy: Recent findings and new physiopathology hypothesis. Prog Retin Eye Res.

[31] He G (2011). Role of CLIC4 in the host innate responses to bacterial lipopolysaccharide. Eur J Immunol.

[32] Ogawa S (2005). Molecular determinants of crosstalk between nuclear receptors and toll-like receptors. Cell.

[33] Bohman S (2005). Proteomic analysis of vascular endothelial growth factor-induced endothelial cell differentiation reveals a role for chloride intracellular channel 4 (CLIC4) in tubular morphogenesis. J Biol Chem.

[34] Chen L (2004). Light damage induced changes in mouse retinal gene expression. Exp Eye Res.

[35] Genis L, Galvez BG, Gonzalo P, Arroyo AG (2006). MT1-MMP: universal or particular player in angiogenesis?. Cancer Metastasis Rev.

[36] Jenkins G (2008). The role of proteases in transforming growth factor-beta activation. Int J Biochem Cell Biol.

[37] Lehmann W (2005). Tumor necrosis factor alpha (TNF-alpha) coordinately regulates the expression of specific matrix metalloproteinases (MMPS) and angiogenic factors during fracture healing. Bone.

[38] Chuang JZ, Chou SY, Sung CH (2010). Chloride Intracellular channel 4 Is critical for the epithelial morphogenesis of RPE cells and retinal attachment. Mol Biol Cell.

[39] Sethi CS, Bailey TA, Luthert PJ, Chong NH (2000). Matrix metalloproteinase biology applied to vitreoretinal disorders. The British journal of ophthalmology.

[40] Curcio CA (2013). Subretinal drusenoid deposits in non-neovascular age-related macular degeneration: morphology, prevalence, topography, and biogenesis model. Retina.

[41] Zarbin MA, Casaroli-Marano RP, Rosenfeld PJ (2014). Age-related macular degeneration: clinical findings, histopathology and imaging techniques. Dev Ophthalmol.

[42] Elliot SJ (2010). Estrogen receptor beta protects against *in vivo* injury in RPE cells. Exp Eye Res.

[43] Alcazar O, Cousins SW, Marin-Castano ME (2007). MMP14 and TIMP-2 overexpression protects against hydroquinone-induced oxidant injury in RPE: implications for extracellular matrix turnover. Invest Ophthalmol Vis Sci.

[44] Elliot S, Catanuto P, Stetler-Stevenson W, Cousins SW (2006). Retinal pigment epithelium protection from oxidant-mediated loss of MMP2 activation requires both MMP14 and TIMP-2. Invest Ophthalmol Vis Sci.

[45] Padgett LC, Lui GM, Werb Z, LaVail MM (1997). Matrix metalloproteinase-2 and tissue inhibitor of metalloproteinase-1 in the retinal pigment epithelium and interphotoreceptor matrix: vectorial secretion and regulation. Exp Eye Res.

[46] Fernandez-Godino R, Bujakowska KM, Pierce EA (2018). Changes in extracellular matrix cause RPE cells to make basal deposits and activate the alternative complement pathway. Hum Mol Genet.

[47] Greene WA, Burke TA, Kaini RR, Por ED, Wang HC (2017). Polarized Secretion of Matrix Metalloproteinases and Their Inhibitors by Retinal Pigment Epithelium Derived from Induced Pluripotent Stem Cells During Wound Healing. J Ocul Pharmacol Ther.

[48] Devy L (2009). Selective inhibition of matrix metalloproteinase-14 blocks tumor growth, invasion, and angiogenesis. Cancer Res.

[49] Argenzio E (2014). CLIC4 regulates cell adhesion and beta1 integrin trafficking. J Cell Sci.

[50] Rozanov DV, Deryugina EI, Monosov EZ, Marchenko ND, Strongin AY (2004). Aberrant, persistent inclusion into lipid rafts limits the tumorigenic function of membrane type-1 matrix metalloproteinase in malignant cells. Exp Cell Res.

[51] Annabi B (2001). Localization of membrane-type 1 matrix metalloproteinase in caveolae membrane domains. Biochem J.

[52] Galvez BG (2004). Caveolae are a novel pathway for membrane-type 1 matrix metalloproteinase traffic in human endothelial cells. Mol Biol Cell.

[53] Mazzone M (2004). Intracellular processing and activation of membrane type 1 matrix metalloprotease depends on its partitioning into lipid domains. J Cell Sci.

[54] Bickel PE (1997). Flotillin and epidermal surface antigen define a new family of caveolae-associated integral membrane proteins. J Biol Chem.

[55] Meister M, Tikkanen R (2014). Endocytic trafficking of membrane-bound cargo: a flotillin point of view. Membranes (Basel).

[56] Hu Y, Chuang JZ, Xu K, McGraw TE, Sung CH (2002). SARA, a FYVE domain protein, affects Rab5-mediated endocytosis. J Cell Sci.

[57] Kajimoto T, Okada T, Miya S, Zhang L, Nakamura S (2013). Ongoing activation of sphingosine 1-phosphate receptors mediates maturation of exosomal multivesicular endosomes. Nat Commun.

[58] Littler DR (2005). Crystal structure of the soluble form of the redox-regulated chloride ion channel protein CLIC4. The FEBS journal.

[59] Meng B, Lever AM (2013). Wrapping up the bad news: HIV assembly and release. Retrovirology.

[60] Babst M (2005). A protein’s final ESCRT. Traffic.

[61] Dozynkiewicz MA (2012). Rab25 and CLIC3 collaborate to promote integrin recycling from late endosomes/lysosomes and drive cancer progression. Dev Cell.

[62] Ponsioen, B. *et al*. Spatiotemporal Regulation of Chloride Intracellular Channel protein CLIC4 by RhoA. *Mol Biol Cell* (2009). 10.1091/mbc.E09-06-0529 PMC2777097 19776349

[63] Bae JS, Yang L, Rezaie AR (2008). Lipid raft localization regulates the cleavage specificity of protease activated receptor 1 in endothelial cells. Journal of Thrombosis and B}Haemostasis: JTH.

[64] Gaus K, Le Lay S, Balasubramanian N, Schwartz MA (2006). Integrin-mediated adhesion regulates membrane order. J Cell Biol.

[65] Fuentes DE, Butler PJ (2012). Coordinated mechanosensitivity of membrane rafts and focal adhesions. Cell Mol Bioeng.

[66] Sobo K (2007). Late endosomal cholesterol accumulation leads to impaired intra-endosomal trafficking. PLoS One.

[67] Sobo K, Chevallier J, Parton RG, Gruenberg J, van der Goot FG (2007). Diversity of raft-like domains in late endosomes. PLoS One.

[68] Smith GA (2016). Extracellular and luminal pH regulation by vacuolar H+-ATPase isoform expression and targeting to the plasma membrane and endosomes. J Biol Chem.

[69] Chung C (2011). The vacuolar-ATPase modulates matrix metalloproteinase isoforms in human pancreatic cancer. Lab Invest.

[70] Rozhin J, Sameni M, Ziegler G, Sloane BF (1994). Pericellular pH affects distribution and secretion of cathepsin B in malignant cells. Cancer Res.

[71] Eisenach PA, de Sampaio PC, Murphy G, Roghi C (2012). Membrane type 1 matrix metalloproteinase (MT1-MMP) ubiquitination at Lys581 increases cellular invasion through type I collagen. J Biol Chem.

[72] Blot V (2004). Nedd4.1-mediated ubiquitination and subsequent recruitment of Tsg101 ensure HTLV-1 Gag trafficking towards the multivesicular body pathway prior to virus budding. J Cell Sci.

[73] Pu J, Guardia CM, Keren-Kaplan T, Bonifacino JS (2016). Mechanisms and functions of lysosome positioning. J Cell Sci.

[74] Freed EO (2002). Viral late domains. Journal of virology.

[75] Baietti MF (2012). Syndecan-syntenin-ALIX regulates the biogenesis of exosomes. Nat Cell Biol.

[76] Dores MR (2012). ALIX binds a YPX(3)L motif of the GPCR PAR1 and mediates ubiquitin-independent ESCRT-III/MVB sorting. J Cell Biol.

[77] Kowal J (2016). Proteomic comparison defines novel markers to characterize heterogeneous populations of extracellular vesicle subtypes. Proc Natl Acad Sci USA.

[78] Jiang L (2012). Intracellular chloride channel protein CLIC1 regulates macrophage function through modulation of phagosomal acidification. J Cell Sci.

[79] McNiven MA (2013). Breaking away: matrix remodeling from the leading edge. Trends Cell Biol.

[80] Samuel W (2017). Appropriately differentiated ARPE-19 cells regain phenotype and gene expression profiles similar to those of native RPE cells. Mol Vis.

[81] Finnemann SC (2003). Focal adhesion kinase signaling promotes phagocytosis of integrin-bound photoreceptors. Embo J.

[82] Finnemann SC, Bonilha VL, Marmorstein AD, Rodriguez-Boulan E (1997). Phagocytosis of rod outer segments by retinal pigment epithelial cells requires alpha(v)beta5 integrin for binding but not for internalization. Proc Natl Acad Sci USA.

[83] Ishikawa M, Sawada Y, Yoshitomi T (2015). Structure and function of the interphotoreceptor matrix surrounding retinal photoreceptor cells. Exp Eye Res.

[84] Wang Z, Juttermann R, Soloway PD (2000). TIMP-2 is required for efficient activation of proMMP2 *in vivo*. J Biol Chem.

[85] Yu HG (2008). Increased choroidal neovascularization following laser induction in mice lacking lysyl oxidase-like 1. Invest Ophthalmol Vis Sci.

[86] Kozhevnikova OS, Korbolina EE, Ershov NI, Kolosova NG (2013). Rat retinal transcriptome: effects of aging and AMD-like retinopathy. Cell Cycle.

[87] Sergejeva O, Botov R, Liutkeviciene R, Kriauciuniene L (2016). Genetic factors associated with the development of age-related macular degeneration. Medicina (Kaunas).

[88] Gambril JA (2019). Quantifying retinal pigment epithelium dysmorphia and loss of histologic autofluorescence in age-related macular degeneration. Invest Ophthalmol Vis Sci.

[89] Casaroli-Marano RP, Pagan R, Vilaro S (1999). Epithelial-mesenchymal transition in proliferative vitreoretinopathy: intermediate filament protein expression in retinal pigment epithelial cells. Invest Ophthalmol Vis Sci.

[90] Spaide RF, Ooto S, Curcio CA (2018). Subretinal drusenoid deposits AKA pseudodrusen. Surv Ophthalmol.

[91] Curcio CA, Johnson M, Rudolf M, Huang JD (2011). The oil spill in ageing Bruch membrane. The British journal of ophthalmology.

[92] Mittelbrunn M (2011). Unidirectional transfer of microRNA-loaded exosomes from T cells to antigen-presenting cells. Nat Commun.

[93] Nabhan JF, Hu R, Oh RS, Cohen SN, Lu Q (2012). Formation and release of arrestin domain-containing protein 1-mediated microvesicles (ARMMs) at plasma membrane by recruitment of TSG101 protein. Proc Natl Acad Sci USA.

[94] Maminishkis A (2006). Confluent monolayers of cultured human fetal retinal pigment epithelium exhibit morphology and physiology of native tissue. Invest Ophthalmol Vis Sci.

[95] Li, Y. *et al*. Patient-specific mutations impair BESTROPHIN1’s essential role in mediating Ca(2+)-dependent Cl(−) currents in human RPE. *eLife* **6**, 10.7554/eLife.29914 (2017). 10.7554/eLife.29914 PMC5655127 29063836

[96] Artym VV, Zhang Y, Seillier-Moiseiwitsch F, Yamada KM, Mueller SC (2006). Dynamic interactions of cortactin and membrane type 1 matrix metalloproteinase at invadopodia: defining the stages of invadopodia formation and function. Cancer Res.

[97] Goldberg AF, Moritz OL, Molday RS (1995). Heterologous expression of photoreceptor peripherin/rds and Rom-1 in COS-1 cells: assembly, interactions, and localization of multisubunit complexes. Biochemistry.

[98] Lingwood D, Simons K (2007). Detergent resistance as a tool in membrane research. Nat Protoc.

[99] Théry Clotilde, Amigorena Sebastian, Raposo Graça, Clayton Aled (2006). Isolation and Characterization of Exosomes from Cell Culture Supernatants and Biological Fluids. Current Protocols in Cell Biology.

