# Supplementary material for: CLIC4 regulates late endosomal trafficking and matrix degradation activity of MMP14 at focal adhesions in RPE cells
Source: Sci Rep. 2019 Aug 22;9:12247. doi: 10.1038/s41598-019-48438-0 (PMC6706427; doi:10.1038/s41598-019-48438-0)
Supplement: Supplementary file 1 — Supplementary Materials [file 41598_2019_48438_MOESM1_ESM.pdf]

SUPPLEMENTARY MATERIALS

CLIC4 regulates late endosomal trafficking and matrix degradation activity of MMP14 at focal adhesions in RPE cells

Kuo-Shun Hsu[1*@], Wataru Otsu[1**@], Yao Li[2], Heuy-Ching Wang[3], Shuibing Chen[4], Stephen H Tsang[2,5,6,7], Jen-Zen Chuang[1], Ching-Hwa Sung[1,8,#]

[1]Department of Ophthalmology, Weill Medical College of Cornell University, New York, NY. [2] Department of Ophthalmology, Columbia University, New York, NY. [3]Ocular Trauma Task Area, US Army Institute of Surgical Research, Joint Base San Antonio-Fort Sam Houston, TX. [4]Department of Surgery and Department of Biochemistry, Weill Medical College of Cornell University. New York, NY. [5]Institute of Human Nutrition, Vagelos College of Physicians and Surgeons, Columbia University, New York, NY. [6] Jonas Children's Vision Care and Bernard & Shirlee Brown Glaucoma Laboratory, Edward S. Harkness Eye Institute, New York-Presbyterian Hospital, New York, NY. [7] Department of Pathology & Cell Biology, and Columbia Stem Cell Initiative, Columbia University Medical Center, New York, NY. [8]Department of Cell and Developmental Biology, Weill Medical College of Cornell University, New York, NY

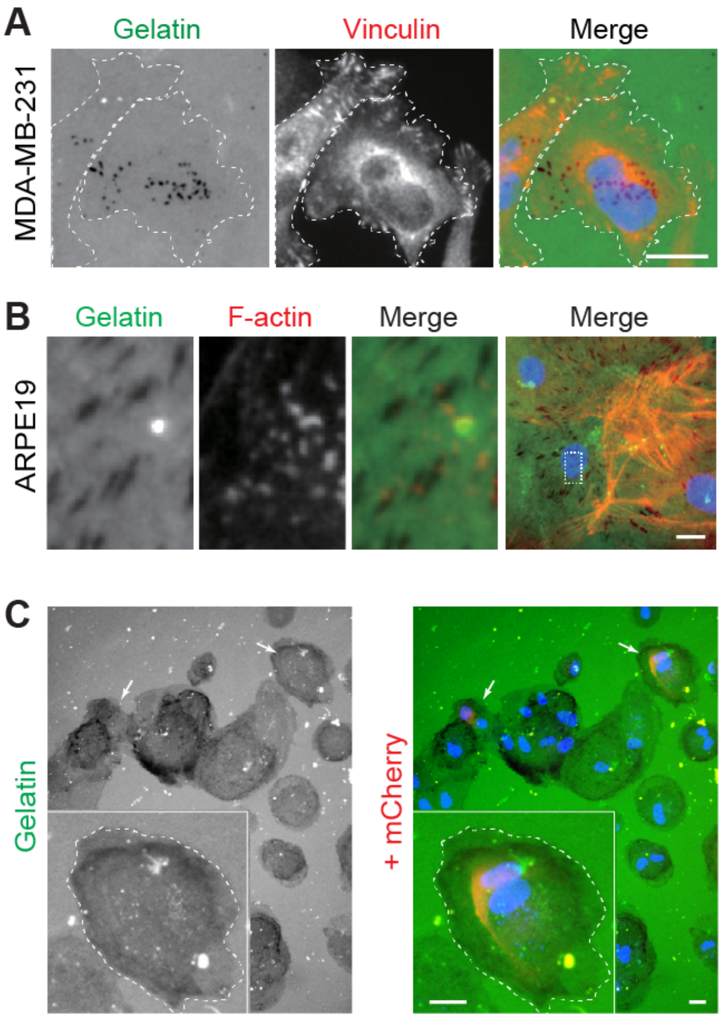

Supplementary Figure 1. The gelatin degradation foci in MDA-MB-231, ARPE19 cells and mCherry overexpressing APRE19 cells. MDA-MB-231 cancer cells (A), APRE19 cells (B), and ARPE19 cells transfected with mCherry alone for 3 days (C) were plated on a fluorescein-conjugated gelatin coverslip for 5-6 hours, fixed, and stained for vinculin (red in A), F-actin (using Alexa594-phalloidin in B), and/or nuclei (blue in A, B, and C). Representative single-color views (in black-and-white) and color-merged images taken by the 20x lens are shown. Dashed lines mark the cell borders (in A, and C). (A) The degradation foci in MDA-MB-231 cells resembled the characteristic invadopodia, which did not look like the vinculin-labeled focal adhesions. (B) The enlarged views of the boxed area in the low-power view (far right panel) showed that the weakly labeled F-actin puncta did not share any specific association with the oblong-shape degradation foci. (C) The low-power views show that the gelatin degradation activity of the mCherry transfected cells (arrows) falls within the normal range of degradation activity of the non-transfected cells. (Inset) The enlarged view of a mCherry expressing cell had bright red fluorescence signals in part of the cell. Scale bars = 10 µm.

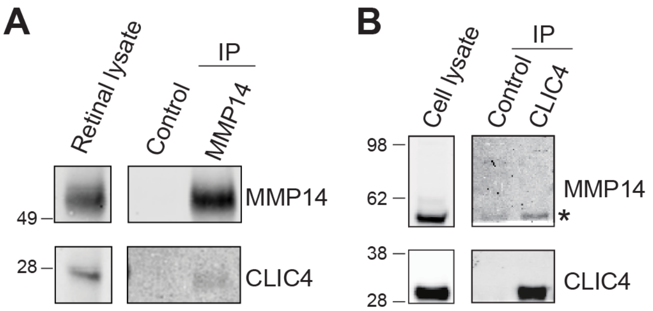

Supplementary Figure 2. Interaction analysis of endogenous CLIC4 and MMP14. (A) Representative blots show that CLIC4 was specifically co-immunoprecipitated with MMP14 from the mouse retinal lysates using anti-MMP14 antibody. (B) Representative blots showed that MMP14 (asterisk) was specifically co-immunoprecipitated with CLIC4 by anti-CLIC4 antibody from the ARPE19 cell lysates. Input represents 5% of the total protein extracts used for immunoprecipitation (IP). The regions containing the immunoglobin heavy chains were trimmed away to avoid the cross-reacted signals from the secondary antibodies.

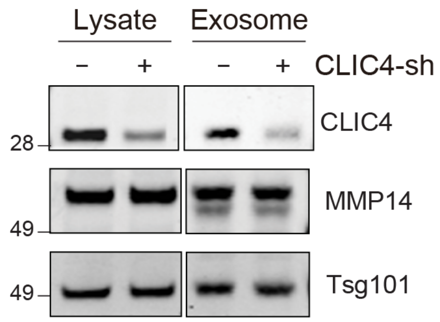

Supplementary Figure 3. Knockdown of CLIC4 did not affect the total exosome expression of MMP14 in ARPE19 cells. Exosomes isolated from the APRE19 cell lines expressing with (+) or without (-) inducible CLIC4-sh and the corresponding total cell lysates (10 µg protein each) were analyzed by immunoblotting with the indicated antibodies.

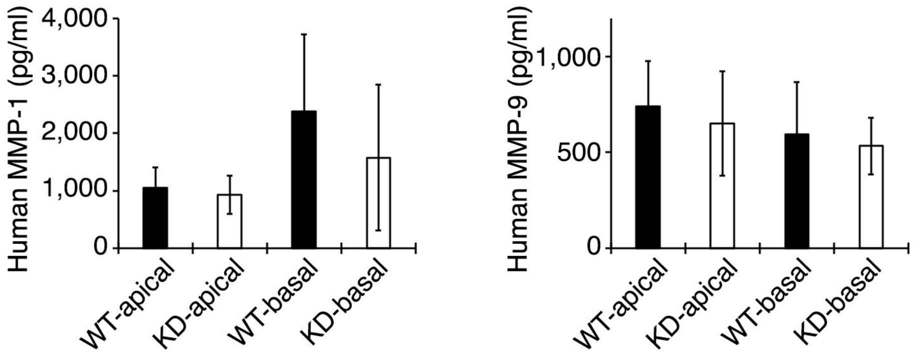

Supplementary Figure 4 CLIC4 KD did not affect the MMP1 and MMP9 secretion in polarized human RPE monolayers. Conditioned media collected from the filter-grown human RPE monolayers expressing the Tet-regulated CLIC4-shRNA were treated without (WT) or with Dox (KD) were subjected to ELISA of MMP1 and MMP9. Bars show means ± S.D. N=3. t-test: no significance.

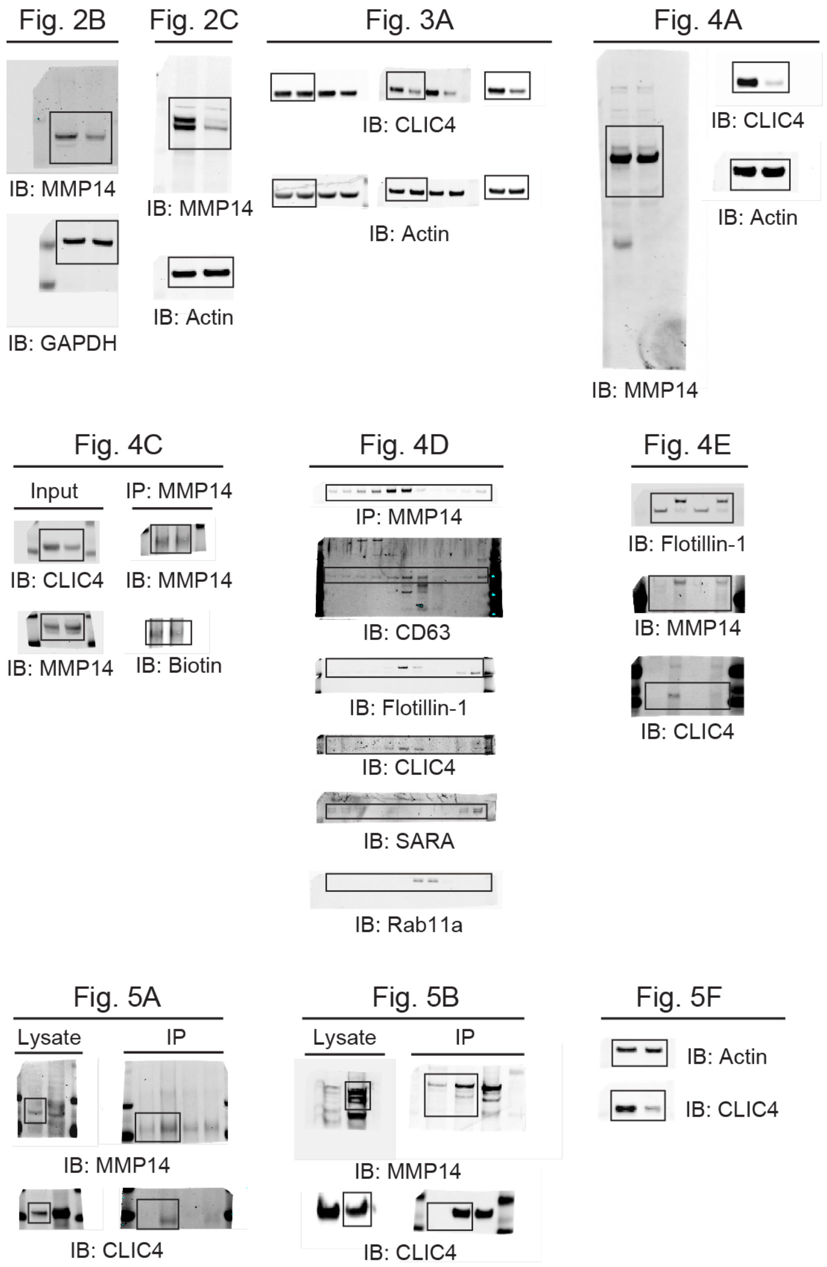

Supplementary Fig. 5. Full scans of immunoblots. The boxed areas are presented in the indicated figures.

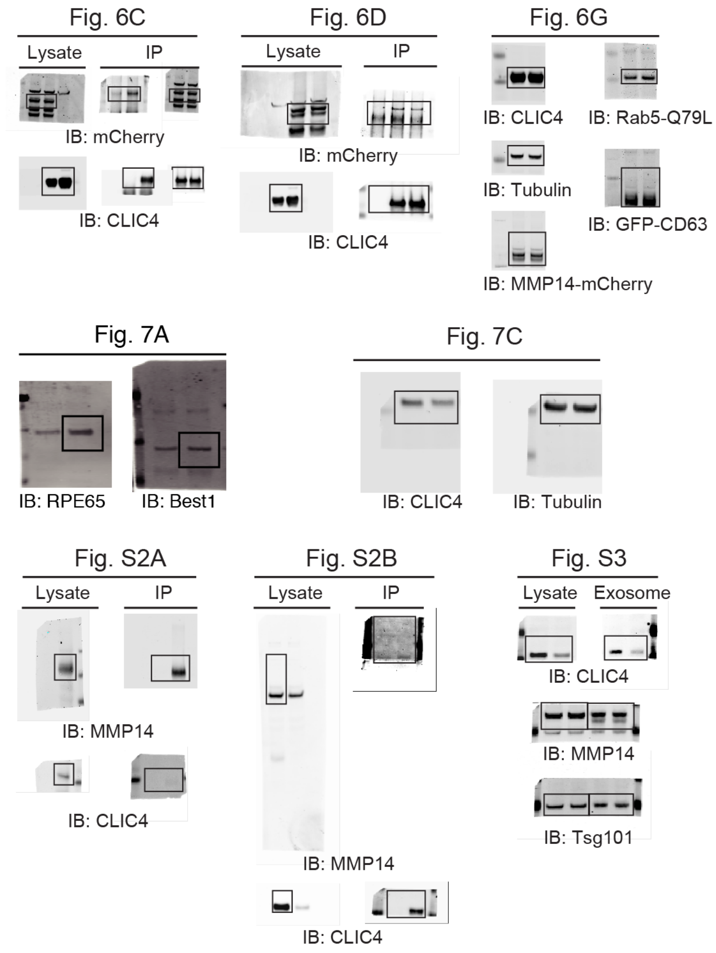

Supplementary Fig. 6. Full scans of indicated immunoblots. The boxed areas are presented in the indicated figures.

Supplementary Movie 1. CLIC4 is expressed in FITC-dextran filled LE in ARPE19 cells Supplemental data to accompany Figure 5E. mCh-CLIC4 expressed ARPE19 cells were incubated with FITC-dextran overnight and chased for 3-hours before recording. Note that CLIC4 and dextran-labeled LE are colocalized and co-migrated. Scale bar = 10 μm.
